# Fitness Level Influences White Matter Microstructure in Postmenopausal Women

**DOI:** 10.3389/fnagi.2020.00129

**Published:** 2020-05-29

**Authors:** Diana Harasym, Claudia V. Turco, Chiara Nicolini, Stephen L. Toepp, E. Madison Jenkins, Martin J. Gibala, Michael D. Noseworthy, Aimee J. Nelson

**Affiliations:** ^1^School of Biomedical Engineering, McMaster University, Hamilton, ON, Canada; ^2^Imaging Research Center, St. Joseph’s Healthcare, Hamilton, ON, Canada; ^3^Department of Kinesiology, McMaster University, Hamilton, ON, Canada; ^4^Department of Electrical and Computer Engineering, McMaster University, Hamilton, ON, Canada; ^5^Department of Radiology, McMaster University, Hamilton, ON, Canada

**Keywords:** GABA, Glu, MRS, DTI, TMS, cortical thickness, sensorimotor

## Abstract

Aerobic exercise has both neuroprotective and neurorehabilitative benefits. However, the underlying mechanisms are not fully understood and need to be investigated, especially in postmenopausal women, who are at increased risk of age-related disorders such as Alzheimer’s disease and stroke. To advance our understanding of the potential neurological benefits of aerobic exercise in aging women, we examined anatomical and functional responses that may differentiate women of varying cardiorespiratory fitness using neuroimaging and neurophysiology. A total of 35 healthy postmenopausal women were recruited (59 ± 3 years) and cardiorespiratory fitness estimated (22–70 mL/kg/min). Transcranial magnetic stimulation was used to assess -aminobutyric acid (GABA) and glutamate (Glu) receptor function in the primary motor cortex (M1), and magnetic resonance spectroscopy (MRS) was used to quantify GABA and Glu concentrations in M1. Magnetic resonance imaging was used to assess mean cortical thickness (MCT) of sensorimotor and frontal regions, while the microstructure of sensorimotor and other white matter tracts was evaluated through diffusion tensor imaging. Regression analysis revealed that higher fitness levels were associated with improved microstructure in pre-motor and sensory tracts, and the hippocampal cingulum. Fitness level was not associated with MCT, MRS, or neurophysiology measures. These data indicate that, in postmenopausal women, higher cardiorespiratory fitness is linked with preserved selective white matter microstructure, particularly in areas that influence sensorimotor control and memory.

## Introduction

Physical activity is associated with improved health and longevity and reduced risk of chronic disease (Warburton and Bredin, 2016). Long-term aerobic exercise is also linked with improved learning, memory and overall brain function, and delayed age-related cognitive decline (Colcombe and Kramer, 2003; Ahlskog et al., 2011; Erickson et al., 2011). Additionally, exercise accelerates rehabilitation in stroke patients and may mitigate symptoms in dementia and Alzheimer’s disease (Heyn et al., 2004; Gallanagh et al., 2011). Previous research focusing on brain effects of long-term exercise has included both males and females. However, considering the greater prevalence of Alzheimer’s disease in women (Mielke et al., 2014; Ferretti et al., 2018) and more profound stroke-related disability experienced by women compared to men (Persky et al., 2010), it is prudent to explore mechanisms by which aerobic exercise prevents or reduces age-related diseases in women.

Research exploring the role of exercise in brain health of postmenopausal women has shown that higher cardiorespiratory fitness correlates with better performance on cognitive assessments (Yaffe et al., 2001; Weuve et al., 2004; Erickson et al., 2007; Brown et al., 2010; Albinet et al., 2014; Dupuy et al., 2015), in addition to greater pre-frontal cortex (PFC) activation during tasks probing executive function (Albinet et al., 2014; Dupuy et al., 2015). These fitness-related effects may be related to structural changes in the brain. For example, fit postmenopausal women have greater cortical volume in the PFC and subgenual cortex compared to their low-fit counterparts (Erickson et al., 2007). High fitness levels in women are also associated with enhanced pre-frontal white matter (WM) tracts (Erickson et al., 2007). Evidence of lower resting mean arterial pressure and greater cerebrovascular conductance in the carotid arteries of fit older women (Brown et al., 2010) is also suggestive of improved brain blood flow.

There remain several unanswered questions regarding the effects of long-term aerobic exercise on the aging female brain. Gains in fitness have been reported to yield improvements in multiple tracts, including the corpus callosum, cingulum, corona radiata, and superior longitudinal fasciculus (Marks et al., 2011; Johnson et al., 2012; Tseng et al., 2013; Oberlin et al., 2016). However, the microstructure of WM tracts, as assessed by a magnetic resonance (MR) technique called diffusion tensor imaging (DTI), has not been explored in postmenopausal women. Neurotransmission, which plays a key role in neural function, has also never been investigated with respect to fitness in aging women. Magnetic resonance spectroscopy (MRS), another MR technique, is able to measure the concentration of neurometabolites in the central nervous system, thus allowing us to determine whether concentrations of the most prevalent inhibitory neurotransmitter, γ-aminobutyric acid (GABA), and excitatory neurotransmitter, glutamate (Glu), change as a function of fitness level in females. Further, transcranial magnetic stimulation (TMS) allows us to indirectly assess whether fitness has an effect on GABAergic and glutamatergic neurotransmitter receptor function in multiple corticospinal circuits. These methods measure different aspects of neurotransmission and whether a relationship exists between these modalities remain unclear (Stagg et al., 2011; Tremblay et al., 2013; Dyke et al., 2017; Mooney et al., 2017; Hermans et al., 2018).

The goal of the present study was to provide a comprehensive description of the neurophysiological and structural differences between postmenopausal women of varying cardiorespiratory fitness. This would enable us to discover potential fitness-related biomarkers associated with overall brain health in this population. Neurophysiological measures were assessed using both TMS and MRS techniques to investigate the primary neurotransmitters, GABA and Glu, in the sensorimotor cortex. Structural measures were assessed with standard anatomical MR images (MRI) and DTI, primarily in the sensorimotor cortex and regions showing an association with fitness in previous literature. An improved understanding of how aerobic exercise and cardiorespiratory fitness achieve their benefits will aid in the design of effective training programs to help combat aging-related disease in females.

## Materials and Methods

### Participants

A total of thirty-five healthy postmenopausal females (59 ± 3 years) participated in the study with a subset of twenty-four individuals included in the MRI portion of the research. Participants provided informed written consent approved by the Hamilton Integrated Research Ethics Board (HiREB). Exclusion criteria included significant medical conditions such as chronic pain, epilepsy, Crohn’s disease, Celiac disease or diabetes, history of head injuries, neurological or psychological disorders, smoking or certain medications (including hormone replacement therapy). Permitted medications included Levothyroxine (*N* = 8), Risedronate (*N* = 3), Rosuvastatin (*N* = 2), Candesartan cilexetil (*N* = 1), Hydrochlorothiazide (*N* = 1), Indapamide (*N* = 1), Perindopril (*N* = 1), Tocilizumab (*N* = 1), and Lansoprazole (*N* = 1). All participants were right-handed as determined by a modified version of the Edinburgh Handedness Scale (Oldfield, 1971). Participants were considered postmenopausal if they had not had a menstrual period in the past 12 months (Al-Safi and Santoro, 2000). The Montreal Cognitive Assessment (MOCA) was administered to ensure participants did not have any symptoms of mild-cognitive impairment (Nasreddine et al., 2005). Participants were included only if their MOCA scores were greater than 26. Physical activity as a rate of energy expenditure (MET-min/week) was determined using the International Physical Activity Questionnaire (IPAQ), which evaluates physical activity over the past week (Craig et al., 2003). Body composition was determined by measuring fat-free body mass and percent body fat (%BF) via air-displacement plethysmography (Bod Pod^®^; Life Measurement Inc., Concord, CA, United States) (Ball, 2005). Participants were asked to fast for 3 h and not to perform any strenuous exercise 12 h prior to the body composition assessment. Thoracic gas volume was estimated for each body composition test using the algorithm provided in the Bod Pod^®^ software.

Cardiorespiratory fitness was assessed for each participant by estimating maximal oxygen consumption (VO_2__max_) in mL/kg/min using a modified version of the submaximal Astrand-Ryhming protocol (Åstrand and Ryhming, 1954; Siconolfi et al., 1982; Pescatello et al., 2014), performed on an electronically braked cycle ergometer (Kettler Ergo Race I; Ense, Germany). Participants were cleared to participate in the fitness test using the Canadian Society for Exercise Physiology (CSEP) Get Active Questionnaire (GAQ). The protocol was initiated with a 1-min warm up at a workload of 25–50W, followed by a 6-min single stage of continuous cycling (60RPM) at a workload of 50–75W, which was designed to increase the heart rate (HR) of participants to a target of 60% of their heart rate reserve (HRR). HRR was estimated for a given individual based on the difference between age-predicted maximal HR (220-age) and resting HR (HR_resting_), as determined by palpation of the radial pulse upon waking and before rising on the morning of the exercise session. Target heart rate was determined using the formula:

$$H\,R_{t\,a\,r\,g\,e\,t}=0.6\times \left(H\,R\,R\right)=0.6\times \left(H\,R_{m\,a\,x}-H\,R_{r\,e\,s\,t\,i\,n\,g}\right)$$

If HR_target_ was less than 120BPM for a participant it was changed to 120BPM, a minimum requirement for the accurate estimation of VO_2__max_ using the Astrand-Ryhming test. Measurements were taken at the end of every minute for at least 6-min using a HR monitor (Polar A3; Lake Success, NY, United States) fitted prior to the fitness assessment. If the target HR was not reached by the 2- and 4-min mark, the workload was adjusted accordingly. At the 6-min mark, if the participant had not reached steady-state at the target HR (i.e., within 5BPM), the intensity was again adjusted and 2 min added to the test. This step was repeated until participants reached the target HR at steady-state for two consecutive minutes. The HR and workload at the last 2 min of the test, along with the participant’s body mass, sex, and age, were used to estimated VO_2__max_ using the Astrand-Ryhming nomogram (Macsween, 2001; Pescatello et al., 2014). Maximal oxygen consumption was also adjusted for body composition (VO_2__max_^*ADJ*^) by using fat-free body mass instead of total mass (Ekelund et al., 2004). This was to account for the possible covariance with obesity (Gazdzinski et al., 2010; Ho et al., 2011; Marks et al., 2011; Dekkers et al., 2019).

### Electrophysiological Acquisition

**FIGURE 1:**
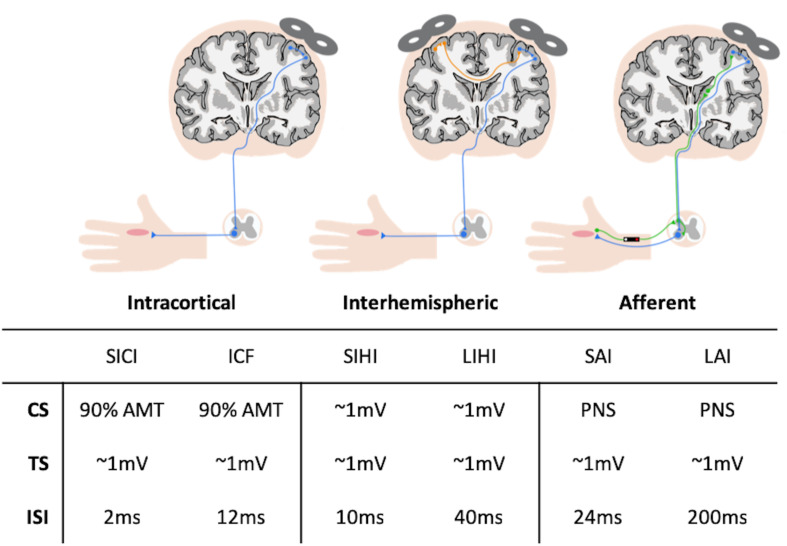
Summary of transcranial magnetic stimulation (TMS) circuitry assessed and parameters used during stimulation. AMT, active motor threshold; CS, conditioning stimulus; ICF, intracortical facilitation; ISI, inter-stimulus interval; SAI, short-latency afferent inhibition; SICI, short-interval intracortical inhibition; SIHI, short-latency interhemispheric inhibition; TS, test stimulus; LAI, long-latency afferent inhibition; LIHI, long-latency interhemispheric inhibition; PNS, peripheral nerve stimulation.

Electrophysiological measures were acquired from all participants 23 ± 14 days from the initial cardiorespiratory fitness test. Figure 1 provides a summary of the neurophysiology assessed using TMS. Participants were asked to refrain from alcohol consumption and strenuous physical exercise 12 h prior to TMS testing. Electromyography (EMG) was used to record motor evoked potentials (MEPs) over the right and left abductor pollicis brevis (APB) using 9-mm diameter Ag-AgCl surface electrodes in a bipolar belly tendon arrangement. A wet ground electrode was placed around the forearm. EMG recordings were amplified by a gain of ×1000, band-pass filtered (20 Hz–2.5 kHz) (Intronix Technologies Corporation Model 2024F with Signal Conditioning; Bolton, ON, Canada), then sampled at 5 kHz (Power1401; Cambridge Electronic Design, Cambridge, United Kingdom) and collected using Signal software (Cambridge Electronic Design; v6.02).

Compound muscle action potentials (CMAP) were recorded to determine the maximal M-wave (M_max_). Recordings were acquired from participants that were seated upright in an armchair with their right arm slightly bent resting on a pillow in the supine position. Nerve stimulation was performed with a constant current stimulator (Digitimer DS7AH; Welwyn Garden City, United Kingdom) and a bar electrode placed over the median nerve at the wrist with the cathode proximal evoking a response in the APB using square wave pulses of 0.2 ms. The nerve stimulator intensity was slowly incremented by 1 mA every 5 s, until the peak-to-peak amplitude of the M-wave did not change for three consecutive increments of the nerve stimulator intensity.

To determine maximum voluntary contraction (MVC), participants completed three maximal isometric contractions of the right APB against an immovable structure. Each contraction lasted 5 s with at least 30 s of rest between trials. Amplified and filtered EMG activity was full-wave rectified and fed into an oscilloscope (Tektronix TDS 2004C; Beaverton, OR, United States) to display the magnitude of the contraction to the participant. For a given participant, the greatest maximum EMG activity obtained from the three trials was considered the MVC. The oscilloscope was set to display a horizontal target line representing the maximum EMG voltage at 10% of MVC. The participant then used visual feedback to match their contraction to the target line during the acquisition of active motor threshold (AMT), which is detailed below.

Transcranial magnetic stimulation was performed using a customized figure-of-eight branding coil (50 mm diameter), connected to a Magstim Bistim stimulator (Whitland, United Kingdom). The motor hotspots of the right and left APB muscles were identified in the left and right hemisphere, respectively, as the cortical location that elicited the largest and most consistent MEP in the relaxed APBs. The coil was orientated at a 45° angle from the sagittal plane to induce a posterior-anterior current. Location and orientation of the motor hotspots were digitally registered using the Brainsight Neuronavigation system (Rogue Research, Montreal, QC, Canada). Resting motor threshold (RMT) and AMT were determined at the motor hotspot for the right APB using the maximum-likelihood parameter estimation by sequential testing (ML-PEST) algorithm found in the TMS Motor Threshold Assessment Tool software (MTAT v2.0) (Ah Sen et al., 2017). The motor threshold assessment was run without *a priori* information and was stopped after 20 stimuli (Ah Sen et al., 2017). In the rest condition, a MEP was defined as having a minimum peak-to-peak amplitude of 50 μV in the relaxed right APB (Rossini et al., 2015). In the active condition, a MEP was defined as a minimum of 200 μV peak-to-peak, while participants maintained ∼10% of their MVC in the right APB using visual feedback from an oscilloscope (Rossini et al., 2015). All single-pulse and paired-pulse TMS paradigms were delivered in a randomized fashion at an interval of 5 s.

Motor evoked potentials recruitment curves (RC) were acquired from the right APB at rest by delivering 8 TMS pulses at TMS intensities in increments of 10% between 90 and 200% RMT. Short-interval intracortical inhibition (SICI) and intracortical facilitation (ICF) were assessed using paired-TMS pulses with an interstimulus interval (ISI) of 2 ms (Lulic et al., 2017) and 12 ms (Dyke et al., 2017), respectively. The conditioning stimulus (CS) was set to an intensity of 90% AMT and the test stimulus (TS) was set to evoke MEPs with peak-to-peak amplitudes of ∼1 mV in the right APB at rest. For each of these measures, 15 conditioned stimuli (CS-TS) and 15 unconditioned stimuli (TS) were randomly delivered.

Short- and long-latency afferent inhibition (SAI and LAI) were assessed by delivering a single TMS pulse over the APB hotspot, preceded by nerve stimulation of the median nerve at the wrist with an ISI of 24 ms (SAI) and 200 ms (LAI) (Turco et al., 2018). Nerve stimulation was performed as described above, with the stimulator intensity set to the motor threshold (MT) of the APB muscle, which was defined as the minimum intensity (in mA) that evoked a visible twitch in the APB. The TS was set to evoke MEPs with peak-to-peak amplitudes of ∼1 mV in the right APB at rest. For each circuit, 15 conditioned stimuli using the nerve stimulator (CS-TS) and 15 unconditioned stimuli (TS) were randomly delivered.

Short- and long-latency interhemispheric inhibition (SIHI and LIHI) were assessed by delivering a CS to the left APB motor hotspot and a TS to the right APB hotspot at an ISI of 10 ms (SIHI) and 40 ms (LIHI) (Hermans et al., 2018). Both the CS and TS were set to evoke MEPs with peak-to-peak amplitudes of ∼1 mV in the left and right APB, respectively. For each circuit, 15 conditioned stimuli (CS-TS) and 15 unconditioned stimuli (TS) were randomly delivered.

### Neuroimaging Acquisition

Only a randomly selected subset of 24 participants were included in the MRI portion of the study based on *a priori* sample calculations. All participants were scanned using a 3T GE MR750 scanner with a 32-channel phased-array head coil (General Electric Healthcare, Milwaukee, WI, United States) about 10 ± 15 days after the TMS session. Participants were asked to refrain from alcohol consumption and strenuous physical exercise 12 h prior to the session. Following a routine 3-plane localizer image and ASSET calibration scan for parallel imaging, an axial 3D IR-prepped T1-weighted fast SPoiled GRadient Echo (fSPGR) structural scan was acquired (TI = 450 ms, TR = 7.9 ms, TE = 3.1 ms, flip angle = 12°, matrix = 240 × 240, FoV = 24 cm, slices = 180, slice thickness = 1 mm isotropic).

**FIGURE 2:**
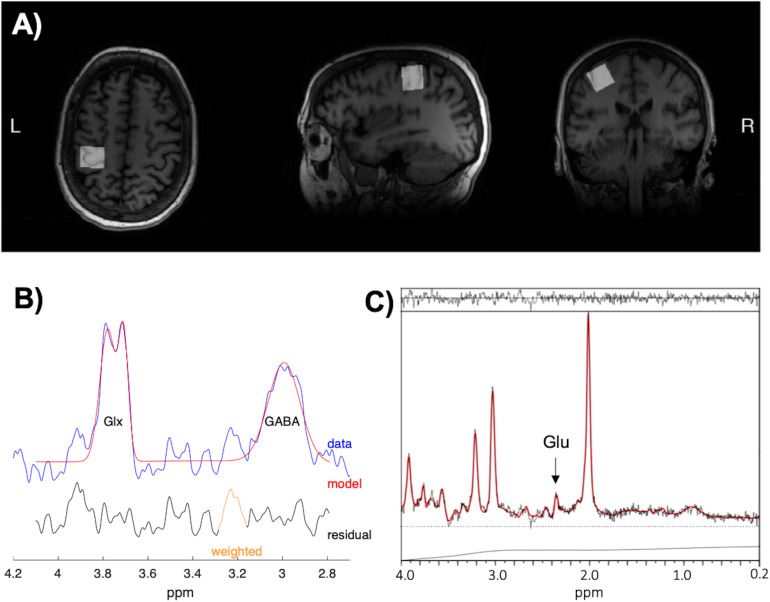
**(A)** The placement of the 20 × 20 × 20 mm voxel of interest (VOI) in the left hand-knob area of the primary motor cortex (M1). The VOI was rotated in the coronal plane to avoid cerebrospinal fluid (CSF) and incorporated the precentral knob and anteriorly directed “hook” along the postcentral gyrus. **(B)** Example spectra and fitting output from Gannet used to measure γ-aminobutyric acid with macromolecule contamination (GABA+) concentrations. **(C)** Example spectra and fitting output from LCModel used to measure glutamate (Glu) concentrations.

The 3D structural scan was used to position a 20 × 20 × 20 mm voxel of interest (VOI) (i.e., 8 cm^3^) over the left hand knob area (M1) with the use of two reliable anatomical landmarks (Yousry et al., 1997). An example of the voxel placement can be seen in Figure 2A, which approximately incorporates the anatomical area stimulated through TMS. The VOI was rotated in the coronal plane to optimize placement in gray matter (GM), avoiding cerebrospinal fluid (CSF). Outer volume suppression (OVS) bands were placed along the outside of the VOI to suppress outer voxel contamination. Automated voxel shimming prior to MRS yielded water-line widths ranging from 3 to 8 Hz. MRS of the VOI was performed to quantify GABA using Mescher–Garwood point-resolved spectroscopy (MEGA-PRESS) GABA editing sequence with water suppression (Mescher et al., 1998) (TR = 2000 ms, TE = 68 ms, number TE’s per scan = 2, number of averages = 320, data points = 2048, receiver bandwidth = 2000 Hz). Additionally, 16 unsuppressed water reference spectra were acquired. Although the Glu composite Glx (Glu + Gln) can be measured reliably from the MEGA-PRESS sequence (Sanaei Nezhad et al., 2018), the acquisition itself was not designed to measure Glu levels. Therefore, Glu concentrations were acquired from the VOI using a stimulated-echo acquisition mode (STEAM) sequence optimized to resolve Glu from GABA and Gln at 3T (TR = 1500 ms, TE = 72 ms, TM = 6 ms, NEX = 8, number of averages = 512, data points = 2048, receiver bandwidth = 5000 Hz) (Sheffield et al., 2009), based on Hu et al. (2007). The STEAM sequence is ideal for coupled spin systems, such as Glu, and allows for better water and macromolecule suppression then PRESS sequences. An example of MEGA-PRESS and optimized STEAM spectra is shown in Figures 2B,C, respectively.

Finally, axial DTI in 60 non-coplanar directions was carried out to assess white matter microstructure using a dual-echo echo-planar imaging (EPI) sequence (TR = 11000 ms, TE = 87 ms, *b*-value = 900 s/mm^2^, matrix = 96 × 96, slices = 50, FOV = 23 cm, slice thickness = 2.9 mm, ASSET = 2). Diffusion was acquired for all 60-directions using 3 separate scans (19, 20, and 21 directions) with three T2-weighted (*b*-value = 0 s/mm^2^) images incorporated at the beginning of each of the acquisitions. Diffusion imaging was acquired last to reduce the effect of frequency offset on MRS measures (Harris et al., 2014).

### Electrophysiological Analysis

**FIGURE 3:**
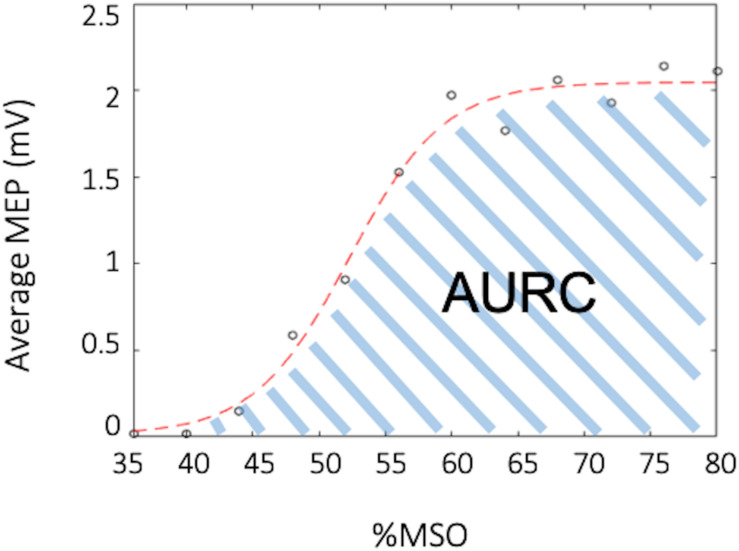
Example recruitment curve (RC). The black circles depict the average muscle evoked potential (MEP) for a specified percent of the maximal stimulator output (%MSO), while the red curve depicts the fitted Boltzmann sigmoid. The area under the recruitment curve (AURC) is calculated as the trapezoidal area under the fitted Boltzmann.

All MEPs were inspected for background muscle activity, and trials were discarded if EMG activity exceeded 50 μV in the 30 ms prior to the TMS stimulus artifact. Data for a given participant was discarded if there were less than 10 trials for paired-pulse paradigms and less than 5 trials for any point on the recruitment curve. For the RC the mean peak-to-peak MEP amplitude was calculated for each %RMT intensity (90–200%). A MATLAB script was then used to fit a Boltzmann sigmoid function to the RC as a percentage of maximal stimulator output (%MSO) using least squares curve-fitting (Carroll et al., 2001; Kukke et al., 2014; Schambra et al., 2015). The function was lower bounded by the minimum noise of the data, which was determined to be 0.013 mV. An example of the fitted curve from one participant can be seen in Figure 3. Area under the RC (AURC) was calculated using trapezoidal integration of the fitted Boltzmann function. Data with an estimated plateau 15% different from the maximum amplitude of the fitted curve were excluded, as this showed that the RC acquired was exponential and had not adequately captured the plateau (Schambra et al., 2015). For paired-pulse TMS measures, the mean peak-to-peak MEP amplitude was calculated for each CS-TS (paired pulse) and TS, separately. The percentage of inhibition and facilitation was determined as a ratio of conditioned over unconditioned stimulus (CS-TS/TS). Inhibition was evident in a circuit when the CS-TS/TS ratio was ≤0.9, while facilitation was evident when the ratio was ≥1.10.

### Neuroimaging Analysis

Magnetic resonance spectroscopy spectra from MEGA-PRESS acquisitions were processed with Gannet (v3.0) using default processing parameters (Edden et al., 2014). GABA concentrations with macromolecule contamination (GABA+) were referenced to water, corrected for frequency offsets, and normalized for macromolecule contamination and editing efficiency (Harris et al., 2015). STEAM spectra were analyzed for Glu with LCModel using basis sets generated through phantom experiments (Provencher, 2001), and were also referenced to water. Tissue composition (%GM, %WM, %CSF) in the VOI was estimated using Gannet by registering the VOI to the T1-weighted anatomical image and segmenting the anatomical image into GM, WM, and CSF using SPM 12 (Ashburner and Friston, 2005). GABA+ and Glu levels were not correlated with tissue content (%Tissue) nor %GM in tissue (%GM_tissue_), hence concentrations were not partial volume corrected (Bogner et al., 2010). The quality of the fitted spectra was assessed by analyzing Cramér-Rao lower bounds (CRLBs) of GABA+ and Glu, which take into account differences in both SNR and line-width of each metabolite (Kreis, 2016). If CRLBs were more than 2SD away from the mean of all participant data for a given measure, the data was excluded from statistical analysis (Kreis, 2016).

**FIGURE 4:**
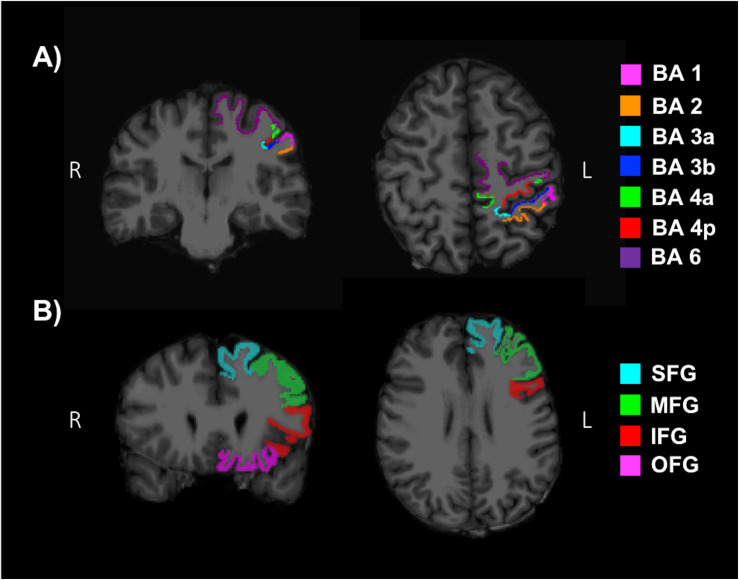
Regions of interests (ROI) used to determine the mean cortical thickness (MCT) in **(A)** the somatosensory (BA 1, 2, 3a, 3b), anterior and posterior primary motor (BA 4a, 4p), and pre-motor (BA 6) regions of the sensorimotor cortex and **(B)** the superior frontal gyrus (SFG), middle frontal gyrus (MFG), inferior frontal gyrus (IFG) and orbitofrontal gyrus (OFG) of the pre-frontal cortex.

T1-weighted images were reconstructed and segmented with the freely available FreeSurfer (v6.0) image analysis suite described in previous literature (Dale and Sereno, 1993; Fischl et al., 1999a, 2004b, 2001, 2002, 2004a, 1999; Fischl and Dale, 2000; Ségonne et al., 2004; Han et al., 2006; Jovicich et al., 2006; Reuter et al., 2010, 2012), using SHARCNET high-performance computing clusters (Shared Hierarchical Academic Research Computing Network and Compute/Calcul Canada). All cortical reconstructions and volumetric segmentations were visually inspected for substantial errors but no manual interventions were applied (McCarthy et al., 2015). Mean cortical thickness (MCT) in the somatosensory (BA 1, 2, 3a, 3b), anterior and posterior M1 (BA 4a, 4p), and pre-motor (BA 6) regions in the left hemisphere were determined by mapping the Brodmann areas and thresholding (Fischl et al., 2008). Additionally, the superior frontal gyrus (SFG), middle frontal gyrus (MFG), inferior frontal gyrus (IFG) and orbitofrontal gyrus (OFG) were evaluated as reference ROIs, since areas of the frontal lobe have commonly shown an increase in volume or MCT with fitness levels (Weinstein et al., 2012; Fletcher et al., 2016; Jonasson et al., 2016; Wood et al., 2016; Williams et al., 2017). A MFG ROI was produced by combining the caudal and rostral middle frontal regions from the Desikan–Killiany anatomical atlas (Fischl et al., 2004b; Desikan et al., 2006). An IFG ROI was produced by combining the pars opercularis, pars triangularis and pars orbitalis. Lastly, an OFG ROI was produced by combining the lateral and medial orbitofrontal regions. Figures 4A,B depict reference ROIs in sensorimotor and prefrontal cortices. MCT was not normalized, since it has not been shown to change as a function of brain size (Im et al., 2008).

**FIGURE 5:**
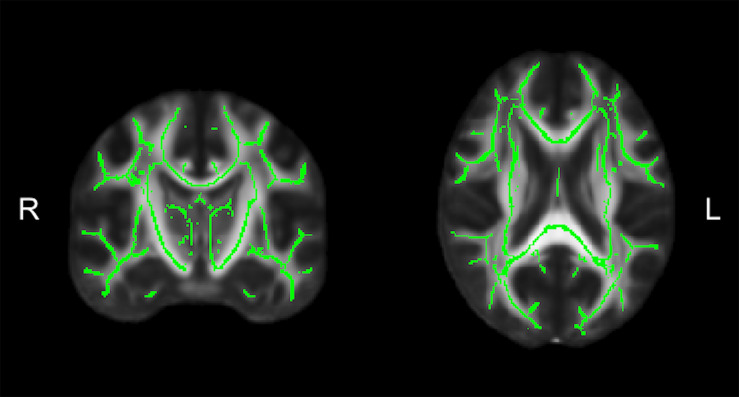
The average white matter (WM) skeleton overlaid on a fractional anisotropic (FA) standard representing WM tracts common to all participants in standard space.

**FIGURE 6:**
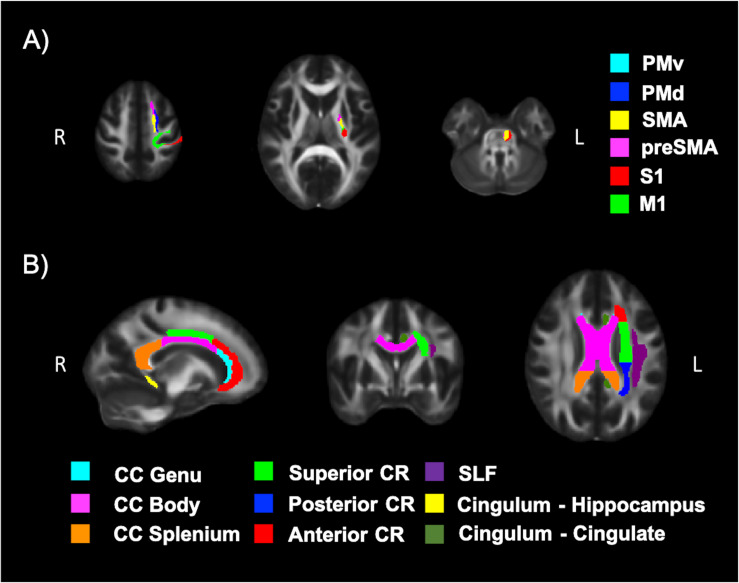
White matter (WM) tracts evaluated using DTI. **(A)** Tracts running between the left corticospinal tract and the left motor cortex (M1), dorsal pre-motor cortex (PMd), ventral pre-motor cortex (PMv), supplementary motor area (SMA), pre-supplementary motor area (preSMA) and primary somatosensory cortex (S1) and **(B)** tracts of the corpus callosum (CC), left corona radiata (CR), cingulum, and superior longitudinal fasciculus (SLF).

Diffusion tensor imaging data was analyzed using the FMRIB Software Library (FSL; v5.0.11) (Jenkinson et al., 2012). Participant data was first skull-stripped using the *Brain Extraction Tool (BET)* (Smith, 2002), then corrected for susceptibility artifacts using B_0_ field maps with *FUGUE*, and finally corrected for eddy-current distortions and head motion using the *eddy_correct* tool (Andersson and Sotiropoulos, 2016). Using *dtifit* (Behrens et al., 2003, 2007) from the FSL Diffusion Toolbox (FDT), diffusion tensors were reconstructed by encoding raw diffusion data as a rank-2 tensor, producing images of fractional anisotropy (FA), axial diffusion (AD), and mean diffusion (MD). Radial diffusion (RD) was calculated using *fslmath* by averaging the second and third eigenvalues. Tract-Based Spatial Statistics (TBSS) was then used to create a WM skeleton representing WM tracts common to all participants in 1 mm^3^ standard Montreal Neurological Institute (MNI152) space using the FMRIB58_FA standard (Smith et al., 2006). The WM skeleton was thresholded at an FA of 0.02 and can be seen overlaid on the FMRIB58_FA standard in Figure 5. Participant DTI were then aligned to this common space using the individualized transformation matrix produced by the non-linear registration tool *FNIRT* in TBSS (Rueckert et al., 1999; Andersson et al., 2007a, b). Each participant’s aligned DTI data was then masked using the WM skeleton, thereby ensuring every participant had diffusion data from WM common to all participants. Next, ROIs on the WM skeleton were selected using the sensorimotor area tract template (SMATT) (Archer et al., 2018), which included tracts running between the corticospinal tract and the left M1, dorsal pre-motor cortex (PMd), ventral pre-motor cortex (PMv), supplementary motor area (SMA), pre-supplementary motor area (preSMA) and primary somatosensory cortex (S1) as shown in Figure 6A. The corpus callosum (CC), left corona radiata (CR), cingulum, and superior longitudinal fasciculus (SLF) from ICBM-DTI-81 white matter labels atlas (Marks et al., 2011; Johnson et al., 2012; Tseng et al., 2013; Oberlin et al., 2016) were also selected as ROI references based on numerous studies that found associations between the WM microstructure of these regions with fitness (Marks et al., 2011; Tseng et al., 2013; Oberlin et al., 2016). A total of 9 reference ROIs were selected and are shown in Figure 6B. Subsequently, average FA, AD, RD and MD were calculated for each ROI in every participant.

### Statistical Analysis

All statistical analyses were carried out using PRISM 6 (GraphPad Software, Inc., v6.0e) and R software [R Core Team (2018), R Foundation for Statistical Computing; v3.5.0]. Normality was assessed using the Shapiro-Wilk test. Outliers were identified using the ROUT method with *Q* = 1% in PRISM (Motulsky and Brown, 2006), which allows for multiple outliers to be detected. Outliers removed from regression modeling and correlations are stated in the footnote of each results table.

Correlation analyses were primarily used to determine if spectroscopic data was unaffected by age and voxel composition to ensure good quality data. Data was analyzed using either Pearson’s product moment correlation coefficient *r* for normally distributed data or Spearman’s rank correlation coefficient ρ for non-parametric data. Correlations between neurotransmitter concentrations and voxel composition (%Tissue and %GM_tissue_) were Bonferroni corrected by a factor of two, where results were considered significant at a threshold of *p* < 0.025. The correlations between water concentrations, which were used as a reference for both GABA+ and Glu, and age had a significance threshold of *p* < 0.05.

Multiple linear regression analysis was used to determine if a relationship existed between neuroimaging and neurophysiological measures and fitness. All dependent measures (Y_d_) were modeled with the following equation:

$$Y_{d}=\beta_{0}+\beta_{F\,i\,t\,n\,e\,s\,s}\,X_{F\,i\,t\,n\,e\,s\,s}+\beta_{A\,g\,e}\,X_{A\,g\,e}+S\,E_{i}$$

where β are the regression coefficients and SE is the standard error associated with fitting the model. Two continuous independent measures (X_i_) were used without any interaction terms, one for fitness (VO_2__max_^ADJ^) and the other to account for age. VO_2__max_^ADJ^ was used instead of VO_2__max_ to correct for body composition differences. All tests were considered significant at a threshold of *p* < 0.05. Both the 95% confidence interval (CI) and the coefficient of determination (*R*^2^) are presented for each model.

## Results

### Cardiorespiratory Fitness

**FIGURE 7:**
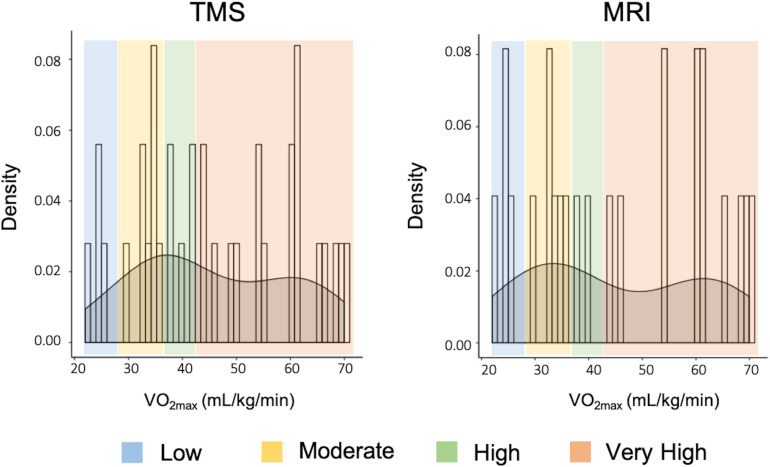
Distribution of VO_2max_ and fitness levels in both TMS and MRI samples.

Demographics and descriptive information of all participants can be found in Table 1. The distribution of VO_2__max_ in both TMS and MRI samples is shown in Figure 7. Both samples had values across a range of fitness levels (low to extremely fit) and within normative values based on the Astrand-Ryhming test in females between the ages of 50–65 years (Astrand, 1960). VO_2__max_ in the TMS sample was normally distributed (*p* = 0.089; skewness = 0.1548, kurtosis = −1.184), while the MRI sample had a non-normal distribution of VO_2__max_ (*p* = 0.047; skewness = 0.1653, kurtosis = −1.469).

**TABLE 1:** Demographics and descriptive information.

	TMS (*N* = 35)	MRI (*N* = 24)
*Age (years)*	59.49 ± 3.40	59.58 ± 3.56
*Vo_2__*max*_ (mL/kg/min)*	45.89 ± 14.26	45.38 ± 15.92
*Vo_2__*max*_^*ADJ*^ (mL/kg/min)*	69.71 ± 14.48	68.50 ± 16.15
*IPAQ (MET/min)*	5412 ± 4635	5406 ± 4135
*%BF*	35.24 ± 9.92	35.17 ± 10.35
*Weight (kg)*	65.42 ± 10.75	68.75 ± 17.00
*Education (years)*	17.17 ± 2.53	17.50 ± 2.64
*MOCA score*	28.14 ± 1.52	28.25 ± 1.48

*Mean ± SD displayed. VO_2max_, maximal oxygen consumption; VO_2max_^*ADJ*^, maximal oxygen consumption adjusted for fat-free body mass; IPAQ, International Physical Activity Questionnaire; %BF, percent body fat; education, full time in years including primary and secondary school; MOCA, Montreal Cognitive Assessment was scored out of 30 possible points.*

### Corticospinal Excitability and Receptor Function

Table 2 shows the regression model for measures of corticospinal excitability and TMS circuits as a factor of fitness and age. One RC was discarded for having less than five trials for multiple points and a total of 4 RCs were discarded for not adequately capturing the plateau. The goodness of fit of the Boltzmann sigmoid to the RC for the remaining participants was adequate with an *R*^2^ of 0.88 ± 0.15. There was no relationship between fitness level and measures of RMT, AMT, AURC, and M_max_. Age was also not significant factor in the model, except for M_max_ which showed an increase with age (*β_*AGE*_* = 0.42, SE = 0.16, *p* = 0.01).

**TABLE 2:** Results of multiple linear regression of corticospinal excitability and TMS circuits against fitness and age.

			Coefficients (β)	SE	95% CI	p-value	R^2^ (Adj.)
*RMT (%MSO)*	*N* = 35	*VO_2__*max*_^*ADJ*^*	0.03	0.11	−0.19:0.25	0.79	0.04
	47.26 ± 9.09	*Age*	0.54	0.46	−0.41:1.48	0.26	
*AMT (%MSO)*	*N* = 35	*VO_2__*max*_^*ADJ*^*	0.13	0.09	−0.04:0.31	0.14	0.03
	34.74 ± 7.40	*Age*	0.38	0.37	−0.37:1.13	0.31	
*M*_*max*_ *(mV)*	*N* = 35	*VO_2__*max*_^*ADJ*^*	0.04	0.04	−0.04:0.11	0.32	0.15
	13.96 ± 3.35	*Age*	0.42	0.16	0.10:0.74	**0.01**	
*AURC*	*N* = 30	*VO_2__*max*_^*ADJ*^*	0.09	0.86	−1.67:1.85	0.91	0.01
	85.83 ± 62.85	*Age*	–1.15	3.38	−8.09:5.79	0.74	
*SICI*	*N* = 35	*VO_2__*max*_^*ADJ*^*	–0.003	0.004	−0.01:0.005	0.41	0.02
	0.52 ± 0.32	*Age*	–0.01	0.02	−0.05:0.02	0.44	
*ICF*	*N* = 34	*VO_2__*max*_^*ADJ*^*	0.007	0.008	−0.009:0.02	0.39	0.04
	1.64 ± 0.67	*Age*	0.01	0.03	−0.06:0.08	0.72	
*SAI*	*N* = 35	*VO_2__*max*_^*ADJ*^*	–0.0003	0.004	−0.009:0.008	0.94	0.04
	0.86 ± 0.35	*Age*	–0.02	0.02	−0.02:0.06	0.27	
*LAI*	*N* = 35	*VO_2__*max*_^*ADJ*^*	–0.002	0.002	−0.008:0.003	0.41	0.02
	0.59 ± 0.25	*Age*	–0.02	0.01	−0.04:0.007	0.15	
*SIHI*	*N* = 35	*VO_2__*max*_^*ADJ*^*	0.0004	0.003	−0.006:0.007	0.90	0.003
	0.52 ± 0.27	*Age*	–0.004	0.01	−0.03:0.02	0.79	
*LIHI*	*N* = 35	*VO_2__*max*_^*ADJ*^*	0.004	0.003	−0.002:0.01	0.21	0.05
	0.64 ± 0.27	*Age*	0.004	0.01	−0.02:0.03	0.78	

*Means ± SD displayed. One outlier removed from ICF. Bolded values indicate significance as shown.*

The depth of inhibition or facilitation of each TMS circuit is depicted in Table 2. There were no significant relationships between fitness or age and each circuit. For intracortical circuits, six participants did not show any facilitation for ICF and 4 participants did not show inhibition for SICI. For interhemispheric circuits, only two participants did not show any SIHI and four participants showed no LIHI. When assessing afferent circuitry, 14 participants showed no SAI and two participants showed no LAI.

### Neurotransmitter Concentrations

One GABA+ and one Glu dataset were poorly fit, with CRLBs 2SDs away from the mean, and hence were discarded. All remaining CRLBs were <20%. Water concentrations were found to be stable across all participants regardless of age (*r* = 0.331, *p* = 0.143). VOI tissue segmentation revealed average %Tissue of 0.83 ± 0.04 and %GM_tissue_ of 0.46 ± 0.09. GABA+ was not correlated with %Tissue (ρ = 0.22, *p* = 0.34) nor %GM_tissue_ (ρ = 0.39, *p* = 0.07). Glu concentrations were also not correlated with %Tissue (ρ = 0.31, *p* = 0.15) nor %GM_tissue_ (ρ = −0.05, *p* = 0.82). Coefficient of variation (%CV) were 19.13% for GABA+ and 9.60% for Glu. All residuals passed normality testing. However, no significant relationship existed between fitness or age and GABA+ or Glu concentrations as seen in Table 3.

**TABLE 3:** Results of multiple linear regression of neurotransmitter concentrations against fitness and age.

			Coefficients (β)	SE	95% CI	p-value	R^2^ (Adj.)
*GABA*+*(mM)*	*N* = 22	*VO_2__*max*_^*ADJ*^*	0.002	0.004	−0.007:0.01	0.66	0.03
	1.61 ± 0.31	*Age*	−0.01	0.02	−0.06:0.03	0.55	
*Glu (mM)*	*N* = 23	*VO_2__*max*_^*ADJ*^*	<0.0001	0.01	−0.02:0.02	0.99	0.03
	8.58 ± 0.82	*Age*	−0.04	0.05	−0.15:0.06	0.42	

*Means ± SD displayed. One outlier removed from GABA+.*

### Cortical Thickness

Fitness did not significantly model MCT in the sensorimotor area nor in the frontal reference ROIs as seen in Table 4.

**TABLE 4:** Results of multiple linear regression of cortical thickness in the sensorimotor area and frontal cortex reference ROIs against fitness and age.

		Coefficients (β)	SE	95% CI	p-value	R^2^ (Adj.)
*BA 1*	*VO_2__*max*_^*ADJ*^*	−0.0001	0.002	−0.004:0.004	0.95	0.08
	*Age*	−0.017	0.009	−0.035:0.001	0.06	
*BA 2*	*VO_2__*max*_^*ADJ*^*	0.0005	0.002	−0.004:0.003	0.80	0.03
	*Age*	−0.006	0.009	−0.024:0.011	0.47	
*BA 3a*	*VO_2__*max*_^*ADJ*^*	0.0008	0.001	−0.002:0.003	0.55	0.03
	*Age*	−0.004	0.006	−0.016:0.009	0.55	
*BA 3b*	*VO_2__*max*_^*ADJ*^*	0.002	0.002	−0.001:0.005	0.23	0.07
	*Age*	0.001	0.007	−0.014:0.017	0.84	
*BA 4a*	*VO_2__*max*_^*ADJ*^*	0.002	0.001	−0.002:0.005	0.31	0.09
	*Age*	−0.012	0.006	−0.025:0.002	0.09	
*BA 4p*	*VO_2__*max*_^*ADJ*^*	0.002	0.002	−0.003:0.007	0.38	0.04
	*Age*	0.001	0.011	−0.022:0.025	0.91	
*BA 6*	*VO_2__*max*_^*ADJ*^*	−0.001	0.001	−0.004:0.002	0.48	0.06
	*Age*	−0.005	0.06	−0.017:0.007	0.39	
*SFG*	*VO_2__*max*_^*ADJ*^*	−0.001	0.001	−0.003:0.001	0.32	0.06
	*Age*	−0.003	0.005	−0.013:0.008	0.63	
*MFG*	*VO_2__*max*_^*ADJ*^*	−0.0001	0.001	−0.002:0.002	0.95	0.08
	*Age*	−0.007	0.005	−0.017:0.003	0.18	
*IFG*	*VO_2__*max*_^*ADJ*^*	−0.0005	0.001	−0.003:0.002	0.70	0.02
	*Age*	−0.003	0.006	−0.015:0.010	0.66	
*OFG*	*VO_2__*max*_^*ADJ*^*	−0.0009	0.001	−0.004:0.002	0.57	0.03
	*Age*	−0.004	0.007	−0.018:0.010	0.53	

### White Matter Microstructure

Fitness was shown to have a significant positive relationship with FA in the PMd (*β_*FITNESS*_* = 0.001, SE = 0.0002, *p* = 0.01) and a significant positive relationship with AD in S1 (*β_*FITNESS*_* = 8.24E-07, SE = 3.76E-07, *p* = 0.04). Regression models of the reference ROIs revealed a significant positive relationship between fitness and AD in the hippocampal cingulum (*β_*FITNESS*_* = 19.19E-07, SE = 6.63E-07, *p* = 0.01). A significant positive relationship between age and RD existed in the preSMA (*β_*AGE*_* = 33.70E-07, SE = 14.85E-07, *p* = 0.03) and S1 (*β_*AGE*_* = 24.98E-07, SE = 10.78E-07, *p* = 0.03) and the cingulum connected to the cingulate gyrus (*β_*AGE*_* = 53.41E-07, SE = 21.48E-07, *p* = 0.02). A detailed summary of all DTI results can be seen in Supplementary Tables S1–S4.

## Discussion

In this study, we investigated the neurophysiological and structural biomarkers that may vary as a function of fitness in postmenopausal women. It is the first study to evaluate WM microstructure in an exclusive cohort of postmenopausal females, and to report that cardiorespiratory fitness is associated with improved WM tracts in pre-motor, sensory and hippocampal areas. Although previous studies have found fitness-related increases in cortical volume in regions of the PFC in postmenopausal women (Erickson et al., 2007), our results did not show an effect of fitness on MCT in the PFC. This study is also the first to evaluate neurophysiological measures in women as a function of fitness and to find no significant effect of fitness on corticospinal excitability or GABAergic and glutamatergic receptor function in the motor cortex. Similarly, we did not observe any significant effect of fitness on the concentration of GABA or Glu within the motor cortex.

We determined that cardiorespiratory fitness was associated with the microstructure of WM tracts traveling between the corticospinal tract and the pre-motor and sensory areas in postmenopausal women. Our results indicated a significant positive relationship between fitness and FA of WM tracts traveling from the corticospinal tract to the left PMd. More fit individuals showed elevated PMd tract FA, suggesting improved microstructure integrity and possibly function (Alexander et al., 2007). Johnson et al. (2012) demonstrated that the majority of WM tracts stemming from a fitness-related region of the CC, connected to the pre-motor cortex. The PMd is associated with motor selection and planning using visual and somatosensory integration, thus playing a critical part in motor initiation and learning (Kantak et al., 2012). One study assessing motor function in aged rhesus monkeys found that faster motor function was associated with greater FA and lower RD in WM tracts contributing to motor processing, such as the internal capsule (Sridharan et al., 2012). Similar results have been seen in humans, where fine finger movement performance has been associated with greater FA and lower MD in the internal capsule (Sullivan et al., 2010). Additionally, 3 weeks of bilateral upper limb training have been shown to increase FA in corticospinal tracts traveling along the internal capsule (Wang et al., 2014). Further, following 5 days of motor learning using the non-dominant hand, the magnitude of skill acquisition was positively related to FA of WM tracts between the ipsilateral PMd and SMA, as well as the contralateral M1 (Schulz et al., 2014). Based on our findings, we suggest that fitness may contribute to improved motor control and learning by preserving pre-motor WM microstructure in postmenopausal women.

In our study, an increase in AD in the left S1 tract was noted in fit individuals, which may indicate reduced axonal loss or degradation of the S1 tract with greater levels of fitness (Alexander et al., 2007). S1 is involved in processing afferent signals and integrating sensory and motor information for the completion of skilled movement (Borich et al., 2015). Previous literature has shown that tactile training for 3 weeks increases FA in the WM of S1 (Debowska et al., 2016). These studies, along with our findings, suggest that cardiorespiratory gains may also yield greater tactile acuity through improved WM microstructure. Further, our findings in the PMd and S1 imply that fitness-related improvements to sensory and pre-motor WM microstructure could promote faster reaction times, as well as better coordination and tactile acuity in postmenopausal women. This is important to consider when it comes to aging, since aging correlates with decreased reaction time, impaired motor coordination (Seidler et al., 2010) and reduced tactile acuity (Kalisch et al., 2009). Promisingly, performing long-term exercise may preserve these functions.

The present study demonstrates that cardiorespiratory fitness is linked to improved WM integrity in tracts associated with memory function in postmenopausal women. Analysis of our reference ROIs reveal an increase in AD as a function of fitness in the portion of the left cingulum connected to the hippocampus, indicating reduced axonal degradation (Alexander et al., 2007). Prior research has also reported an improvement in the microstructure of the cingulum associated with fitness levels (Marks et al., 2011; Tseng et al., 2013; Oberlin et al., 2016). In particular, similar to our study, Tseng et al. (2013) observed that Master athletes have lower MD in the left hippocampal cingulum compared to their sedentary counterparts. The hippocampus plays an important role in memory function and has been consistently implicated in the neural plasticity effects of long-term exercise (Erickson et al., 2014), with larger hippocampal volume associated with greater cardiorespiratory fitness and improved memory (Erickson et al., 2009). Enhanced cingulum microstructure has also been shown to promote memory function (Kantarci et al., 2011). Based on these findings, it is likely that the fitness-related improvements to the microstructure of the hippocampal cingulum observed in our study may also contribute to improving memory function.

Contrary to previous studies reporting that fitness is linked with increased MCT in the left paracentral gyrus (Williams et al., 2017), precentral gyrus, postcentral gyrus, MFG (Wood et al., 2016) and in the dorsolateral PFC (Jonasson et al., 2016), here we found no significant relationship between fitness and MCT in the left PFC and sensorimotor areas in postmenopausal women. This finding is also in contradiction with Erickson et al., who found fitness-related increases in the cortical volume of the PFC in postmenopausal women (Erickson et al., 2007). Although related, cortical volume is not a direct measure of MCT (Winkler et al., 2010), which may explain the differences between our findings. Further, there are differences in the relative fitness of the sample populations, with only one of the studies having a wide range of fitness levels similar to our sample (Wood et al., 2016). Most notable is that we studied only women and previous studies included both sexes.

Our data indicates that cardiorespiratory fitness does not influence corticospinal excitability or GABAergic and glutamatergic receptor function in the motor cortex of postmenopausal women, which is in line with previous findings (Cirillo et al., 2009; McGregor et al., 2011, 2012, 2013, 2017; Lulic et al., 2017). For example, fitness does not change SICI, a measure of intracortical GABAergic receptor function, in the APB (Cirillo et al., 2009) and flexor carpi radialis (FCR) (Lulic et al., 2017). Likewise, intracortical glutamatergic receptor function in the form of ICF in the FCR is unaffected by fitness (Lulic et al., 2017). LIHI, which assesses interhemispheric GABAergic receptor function, is also unaffected by fitness (McGregor et al., 2017). Nonetheless, other *trans-*callosal circuits such as ipsilateral silent period (iSP) increase with fitness and physical activity (McGregor et al., 2011, 2012, 2017). Our study also found that afferent circuitry, specifically SAI and LAI, are not affected by fitness. Thus, taken together, our results further corroborate previous findings demonstrating that corticospinal excitability of the upper limb is unaffected by physical activity and fitness (Cirillo et al., 2009; McGregor et al., 2011, 2012, 2013, 2017; Lulic et al., 2017). Additionally, the fact that M_max_ does not change with fitness suggests that there are no training effects on the muscle composition (muscle fiber concentrations and area) of the APB that might influence EMG recordings (Molin and Punga, 2016). However, this would have to be verified with concurrent muscle MRI and EMG measures (e.g., Akbari et al., 2016).

The current study was the first to evaluate GABA concentrations as a function of fitness. Specifically, we found that GABA+ in the primary motor cortex was unaffected by fitness level in postmenopausal women. Our study does not support previous research showing that higher levels of physical activity predict higher resting Glu levels (Maddock et al., 2016), while more fit individuals have lower resting Glu levels (Dennis et al., 2015). In contrast, it was found that Glu concentrations are unaffected by fitness in postmenopausal women. However, previous studies investigated populations of both men and women, and assessed Glu concentrations in the occipital lobe (Dennis et al., 2015; Maddock et al., 2016), as opposed to examining only females and the motor cortex as we did here. Furthermore, we used an optimized STEAM sequence that better separated the complex splitting patterns of Glx peaks leading to better delineation of glutamate and glutamine (Sheffield et al., 2009).

Age is a co-variate that we and others have included in the regression model (Fletcher et al., 2016; Wood et al., 2016). However, age did not have a significant effect on most of the neurophysiological and MCT measures. This is likely due to the narrow age range of participants (52–65 years) and the fact that this age-range does not reflect the point at which age-related decline becomes more apparent. Nonetheless, age had some influence on structural markers. A significant positive relationship between age and RD existed in the tracts of the left preSMA and S1, as well as in the tracts that connect the left cingulum to the cingulate. This relationship indicates that aging may cause demyelination of axons in these tracts (Alexander et al., 2007), high-lighting the fact that aging interferes with structures associated with both sensorimotor and cognitive function in postmenopausal women.

### Limitations

One of the major limitations of our work is the macromolecule contamination of the GABA+ signal due to the transmit RF bandwidth of the editing pulse in MEGA-PRESS, which may play a role in the observed results (Mullins et al., 2014). Although MEGA-PRESS with macromolecule suppression exists, this method still suffers from high variability of GABA levels due to the influence of frequency drift (Mikkelsen et al., 2017). It is also important to note that measures of GABA+ from the VOI are not strictly from M1, but actually include other areas of the sensorimotor cortex, an effect of partial volume. It is also unknown whether the area being assessed with MRS is the exact same area being assessed with TMS, since the placement of the VOI is based on structural markers, while the location of the TMS hotspot is based on a functional paradigm. Future studies would benefit from collecting MRI data prior to the TMS assessment, and using the acquired anatomical images to ensure stimulation is applied at the exact area where the VOI was placed. An additional limitation is that SAI was not assessed using measured somatosensory evoked potentials (SEPs) to determine an ISI for each individual based on the N20 component. Hence, the depth of inhibition may not be at its maximum.

Diffusion tensor imaging results should also be interpreted with caution as crossing fibers may affect the observed values (Soares et al., 2013). Our results showed no effect of fitness on neurophysiology. However, these observations are limited to the motor cortex, and are not generalizable to other regions of the brain. As the majority of fitness-related changes have been localized to the frontal lobe and hippocampus, it is possible that these areas may show changes in neurotransmission and thus require future investigation.

We would also like to acknowledge the small sample size, although we matched or exceeded the sample size of previous studies (Cirillo et al., 2009; Marks et al., 2011; McGregor et al., 2011, 2012, 2017; Dennis et al., 2015; Maddock et al., 2016; Wood et al., 2016; Lulic et al., 2017). Further, the association between cognitive aptitude and years of education on brain structure should be noted (Gordon et al., 2008; Ho et al., 2011; Kaup et al., 2011; Bennett and Madden, 2014; Cremers et al., 2016; Cacciaglia et al., 2018). Although cognitive tests and education were not used to adjust the data, to ensure participants had no significant cognitive deficits, they were screened using the MOCA, which incorporates years of education as part of the scores. Participants with a MOCA score of 26 might have minimal cognitive impairment. Six participants out of thirty-five had a score of 26, which is, however, the minimum passing score of the MOCA and thus acceptable (Nasreddine et al., 2005).

Lastly, we used a modified version of the Astrand-Ryhming cycling ergometer protocol (Åstrand and Ryhming, 1954; Siconolfi et al., 1982; Pescatello et al., 2014), a commonly used submaximal cardiorespiratory fitness assessment to estimate VO_2__max_ (Guiney et al., 2015; Kawagoe et al., 2017). Estimating VO_2__max_ using a sub-maximal test allowed participants who may be at-risk during a maximal fitness test to be included. However, a maximal cardiorespiratory fitness assessment to directly measure VO_2__max_ could be used in future studies to confirm our findings.

### Conclusions

This was the first study to comprehensively assess neurophysiological and structural markers as a function of fitness in postmenopausal women. Our results indicate that fitness may affect the microstructure of WM tracts associated with both motor control and sensory processing. Further, we show no fitness-related changes to GABA or Glu neurotransmission in the motor cortex of postmenopausal women. Whether these findings can be translated to other regions of the brain remains to be investigated. Future research should probe whether fitness gains are associated with improved motor skill function such as coordination, and tactile perception in postmenopausal women, and last, whether long-term exercise will preserve this sensorimotor function into their senior years.

## Data Availability Statement

The data supporting these findings are available in Mendeley Datasets at http://doi.org/10.17632/fvh7b4hwsg.1.

## Ethics Statement

All studies involving human participants were reviewed and approved by the Hamilton Integrated Research Ethics Board (HiREB). Participants who took part in this study provided their written informed consent.

## Author Contributions

DH designed and performed experiments, analyzed and interpreted the data, and wrote the manuscript. CT, CN, ST, and EJ performed the experiments. MG and MN supervised the experiments. AN funded, directed, and supervised experiments. All authors contributed to manuscript writing as well as read and approved the final manuscript.

## Conflict of Interest

The authors declare that the research was conducted in the absence of any commercial or financial relationships that could be construed as a potential conflict of interest.

## References

[B1] Ah Sen C. B. Fassett H. J. El-Sayes J. Turco C. V. Hameer M. M. Nelson A. J. (2017). Active and resting motor threshold are efficiently obtained with adaptive threshold hunting. *PLoS One* 12:e0186007. 10.1371/journal.pone.0186007 28982146 PMC5628904

[B2] Ahlskog J. E. Geda Y. E. Graff-Radford N. R. Petersen R. C. (2011). Physical exercise as a preventive or disease-modifying treatment of dementia and brain aging. *Mayo Clin. Proc.* 86 876–884. 10.4065/mcp.2011.0252 21878600 PMC3258000

[B3] Akbari A. Rockel C. P. Kumbhare D. A. Noseworthy M. D. (2016). Safe MRI-Compatible electrical muscle stimulation (EMS) system. *J. Magn. Reson. Imaging* 44 1530–1538. 10.1002/jmri.25316 27185587

[B4] Albinet C. T. Mandrick K. Bernard P. L. Perrey S. Blain H. (2014). Improved cerebral oxygenation response and executive performance as a function of cardiorespiratory fitness in older women: a fNIRS study. *Front. Aging Neurosci.* 6:272. 10.3389/fnagi.2014.00272 25339900 PMC4189417

[B5] Alexander A. L. Lee J. E. Lazar M. Field A. S. (2007). Diffusion tensor imaging of the brain. *Neurotherapeutics* 4 316–329. 10.1016/j.nurt.2007.05.011 17599699 PMC2041910

[B6] Al-Safi Z. Santoro N. (2000). “The postmenopausal woman,” in *Endotext*, eds Kenneth (South Dartmouth, MA: MDText.com, Inc.)

[B7] Andersson J. L. R. Jenkinson M. Smith S. (2007a). *Non-linear registration, aka Spatial normalisation FMRIB technical report TR07JA2.* Oxford: FMRIB Analysis Group of the University of Oxford, Vol. 2.

[B8] Andersson J. L. R. Jenkinson M. Smith S. Andersson J. (2007b). *Non-linear optimisation FMRIB Technial Report TR07JA1.* FMRIB Technial Report TR07JA1. Oxford: University of Oxford.

[B9] Andersson J. L. R. Sotiropoulos S. N. (2016). An integrated approach to correction for off-resonance effects and subject movement in diffusion MR imaging. *Neuroimage* 125 1063–1078. 10.1016/j.neuroimage.2015.10.019 26481672 PMC4692656

[B10] Archer D. B. Vaillancourt D. E. Coombes S. A. (2018). A template and probabilistic atlas of the human sensorimotor tracts using diffusion MRI. *Cereb. Cortex* 28 1685–1699. 10.1093/cercor/bhx066 28334314 PMC5907352

[B11] Ashburner J. Friston K. J. (2005). Unified segmentation. *Neuroimage* 26 839–851. 10.1016/j.neuroimage.2005.02.018 15955494

[B12] Astrand I. (1960). Aerobic work capacity in men and women with special reference to age. *Acta Physiol. Scand. Supplement.* 49 1–92. 13794892

[B13] Åstrand P.-O. Ryhming I. (1954). A nomogram for calculation of aerobic capacity (Physical Fitness) from pulse rate during submaximal work. *J. Appl. Physiol.* 7 218–221. 10.1152/jappl.1954.7.2.218 13211501

[B14] Ball S. D. (2005). Interdevice variability in percent fat estimates using the BOD POD. *Eur. J. Clin. Nutr.* 59 996–1001. 10.1038/sj.ejcn.1602202 15970945

[B15] Behrens T. E. J. Berg H. J. Jbabdi S. Rushworth M. F. S. Woolrich M. W. (2007). Probabilistic diffusion tractography with multiple fibre orientations: what can we gain? *Neuroimage* 34 144–155. 10.1016/j.neuroimage.2006.09.018 17070705 PMC7116582

[B16] Behrens T. E. J. Woolrich M. W. Jenkinson M. Johansen-Berg H. Nunes R. G. Clare S. (2003). Characterization and propagation of uncertainty in diffusion-weighted MR imaging. *Magn. Reson. Med.* 50 1077–1088. 10.1002/mrm.10609 14587019

[B17] Bennett I. J. Madden D. J. (2014). Disconnected aging: cerebral white matter integrity and age-related differences in cognition. *Neuroscience* 276 187–205. 10.1016/j.neuroscience.2013.11.026 24280637 PMC4032380

[B18] Bogner W. Gruber S. Doelken M. Stadlbauer A. Ganslandt O. Boettcher U. (2010). In vivo quantification of intracerebral GABA by single-voxel (1)H-MRS-How reproducible are the results? *Eur. J. Radiol.* 73 526–531. 10.1016/j.ejrad.2009.01.014 19201120

[B19] Borich M. R. Brodie S. M. Gray W. A. Ionta S. Boyd L. A. (2015). Understanding the role of the primary somatosensory cortex: opportunities for rehabilitation. *Neuropsychologia* 79(Pt B), 246–255. 10.1016/j.neuropsychologia.2015.07.007 26164474 PMC4904790

[B20] Brown A. D. McMorris C. A. Longman R. S. Leigh R. Hill M. D. Friedenreich C. M. (2010). Effects of cardiorespiratory fitness and cerebral blood flow on cognitive outcomes in older women. *Neurobiol. Aging* 31 2047–2057. 10.1016/j.neurobiolaging.2008.11.002 19111937

[B21] Cacciaglia R. Molinuevo J. L. Sánchez-Benavides G. Falcón C. Gramunt N. Brugulat-Serrat A. (2018). Episodic memory and executive functions in cognitively healthy individuals display distinct neuroanatomical correlates which are differentially modulated by aging. *Hum. Brain Mapp.* 39 4565–4579. 10.1002/hbm.24306 29972619 PMC6220988

[B22] Carroll T. J. Riek S. Carson R. G. (2001). Reliability of the input-output properties of the cortico-spinal pathway obtained from transcranial magnetic and electrical stimulation. *J. Neurosci. Methods* 112 193–202. 10.1016/S0165-0270(01)00468-X 11716954

[B23] Cirillo J. Lavender A. P. Ridding M. C. Semmler J. G. (2009). Motor cortex plasticity induced by paired associative stimulation is enhanced in physically active individuals. *J. Physiol.* 587(Pt 24), 5831–5842. 10.1113/jphysiol.2009.181834 19858227 PMC2808543

[B24] Colcombe S. Kramer A. F. (2003). Fitness effects on the cognitive function of older adults: a meta-analytic study. *Psychol. Sci.* 14 125–130. 10.1111/1467-9280.t01-1-01430 12661673

[B25] Craig C. L. Marshall A. L. Sjostrom M. E. Bauman A. E. Booth M. L. Oja P. (2003). International physical activity questionnaire: 12-country reliability and validity. *Med. Sci. Sports Exerc.* 35 1381–1395. 10.1249/01.MSS.0000078924.61453.FB 12900694

[B26] Cremers L. G. M. de Groot M. Hofman A. Krestin G. P. van der Lugt A. Niessen W. J. (2016). Altered tract-specific white matter microstructure is related to poorer cognitive performance: The Rotterdam Study. *Neurobiol. Aging* 39 108–117. 10.1016/j.neurobiolaging.2015.11.021 26923407

[B27] Dale A. M. Sereno M. I. (1993). Improved localizadon of cortical activity by combining EEG and MEG with MRI cortical surface reconstruction: a linear approach. *J. Cogn. Neurosci.* 5 162–176. 10.1162/jocn.1993.5.2.162 23972151

[B28] Debowska W. Wolak T. Nowicka A. Kozak A. Szwed M. Kossut M. (2016). Functional and structural neuroplasticity induced by short-term tactile training based on braille reading. *Front. Neurosci.* 10:460. 10.3389/fnins.2016.00460 27790087 PMC5061995

[B29] Dekkers I. A. Jansen P. R. Lamb H. J. (2019). Obesity, brain volume, and white matter microstructure at MRI: a cross-sectional UK biobank study. *Radiology* 291 763–771. 10.1148/radiol.2019181012 31012815

[B30] Dennis A. Thomas A. G. Rawlings N. B. Near J. Nichols T. E. Clare S. (2015). An ultra-high field magnetic resonance spectroscopy study of post exercise lactate, glutamate and glutamine change in the human brain. *Front. Physiol.* 6:351. 10.3389/fphys.2015.00351 26732236 PMC4681779

[B31] Desikan R. S. Ségonne F. Fischl B. Quinn B. T. Dickerson B. C. Blacker D. (2006). An automated labeling system for subdividing the human cerebral cortex on MRI scans into gyral based regions of interest. *Neuroimage* 31 968–980. 10.1016/j.neuroimage.2006.01.021 16530430

[B32] Dupuy O. Gauthier C. J. Fraser S. A. Desjardins-Crãpeau L. Desjardins M. Mekary S. (2015). Higher levels of cardiovascular fitness are associated with better executive function and prefrontal oxygenation in younger and older women. *Front. Hum. Neurosci.* 9:66. 10.3389/fnhum.2015.00066 25741267 PMC4332308

[B33] Dyke K. Pepes S. E. Chen C. Kim S. Sigurdsson H. P. Draper A. (2017). Comparing GABA-dependent physiological measures of inhibition with proton magnetic resonance spectroscopy measurement of GABA using ultra-high-field MRI. *Neuroimage* 152 360–370. 10.1016/j.neuroimage.2017.03.011 28284797 PMC5440178

[B34] Edden R. A. Puts N. A. Harris A. D. Barker P. B. Evans C. J. (2014). Gannet: a batch-processing tool for the quantitative analysis of gamma-aminobutyric acid-edited MR spectroscopy spectra. *J. Magn. Reson. Imaging* 40 1445–1452. 10.1002/jmri.24478 25548816 PMC4280680

[B35] Ekelund U. Franks P. W. Wareham N. J. Åman J. (2004). Oxygen uptakes adjusted for body composition in normal-weight and obese adolescents. *Obes. Res.* 12 513–520. 10.1038/oby.2004.58 15044669

[B36] Erickson K. I Prakash R. S. Voss M. W. Chaddock L. Hu L. Morris K. S. (2009). Aerobic fitness is associated with hippocampal volume in elderly humans. *Hippocampus* 19 1030–1039. 10.1002/hipo.20547 19123237 PMC3072565

[B37] Erickson K. I Voss M. W. Prakash R. S. Basak C. Szabo A. Chaddock L. (2011). Exercise training increases size of hippocampus and improves memory. *Proc. Natl. Acad. Sci. U.S.A.* 108 3017–3022. 10.1073/pnas.1015950108 21282661 PMC3041121

[B38] Erickson K. I. Colcombe S. J. Elavsky S. McAuley E. Korol D. L. Scalf P. E. (2007). Interactive effects of fitness and hormone treatment on brain health in postmenopausal women. *Neurobiol. Aging* 28 179–185. 10.1016/j.neurobiolaging.2005.11.016 16406152

[B39] Erickson K. I. Leckie R. L. Weinstein A. M. (2014). Physical activity, fitness, and gray matter volume. *Neurobiol. Aging* 35 S20–S28. 10.1016/j.neurobiolaging.2014.03.034 24952993 PMC4094356

[B40] Ferretti M. T. Iulita M. F. Cavedo E. Chiesa P. A. Schumacher Dimech A. Santuccione Chadha A. (2018). Sex differences in Alzheimer disease — the gateway to precision medicine. *Nat. Rev. Neurol.* 14 457–469. 10.1038/s41582-018-0032-9 29985474

[B41] Fischl B. Dale A. M. (2000). Measuring the thickness of the human cerebral cortex from magnetic resonance images. *Proc. Natl. Acad. Sci. U.S.A.* 97 11050–11055. 10.1073/pnas.200033797 10984517 PMC27146

[B42] Fischl B. Liu A. Dale A. M. (2001). Automated manifold surgery: constructing geometrically accurate and topologically correct models of the human cerebral cortex. *IEEE Trans. Med. Imaging* 20 70–80. 10.1109/42.906426 11293693

[B43] Fischl B. Rajendran N. Busa E. Augustinack J. Hinds O. Yeo B. T. T. (2008). Cortical folding patterns and predicting cytoarchitecture. *Cereb. Cortex* 18 1973–1980. 10.1093/cercor/bhm225 18079129 PMC2474454

[B44] Fischl B. Salat D. H. Busa E. Albert M. Dieterich M. Haselgrove C. (2002). Whole brain segmentation: automated labeling of neuroanatomical structures in the human brain. *Neuron* 33 341–355. 11832223 10.1016/s0896-6273(02)00569-x

[B45] Fischl B. Salat D. H. van der Kouwe A. J. W. Makris N. Ségonne F. Quinn B. T. (2004a). Sequence-independent segmentation of magnetic resonance images. *Neuroimage* 23 S69–S84. 10.1016/j.neuroimage.2004.07.016 15501102

[B46] Fischl B. van der Kouwe A. Destrieux C. Halgren E. Ségonne F. Salat D. H. (2004b). Automatically parcellating the human cerebral cortex. *Cereb. Cortex* 14 11–22. 10.1093/cercor/bhg087 14654453

[B47] Fischl B. Sereno M. I. Dale A. M. Fischl B. Sereno M. I. (1999a). Cortical surface-based analysis. *Neuroimage* 9 179–194. 10.1006/nimg.1998.0395 9931268

[B48] Fischl B. Sereno M. I. Tootell R. B. Dale A. M. (1999b). High-resolution intersubject averaging and a coordinate system for the cortical surface. *Hum. Brain Mapp.* 8 272–284. 10.1002/(sici)1097-0193(1999)8:4<272::aid-hbm10>3.0.co;2-4 10619420 PMC6873338

[B49] Fletcher M. A. Low K. A. Boyd R. Zimmerman B. Gordon B. A. Tan C. H. (2016). Comparing aging and fitness effects on brain anatomy. *Front. Hum. Neurosci.* 10:286. 10.3389/fnhum.2016.00286 27445740 PMC4923123

[B50] Gallanagh S. Quinn T. J. Alexander J. Walters M. R. (2011). Physical activity in the prevention and treatment of stroke. *ISRN Neurol.* 2011:953818. 10.5402/2011/953818 22389836 PMC3263535

[B51] Gazdzinski S. Millin R. Kaiser L. G. Durazzo T. C. Mueller S. G. Weiner M. W. (2010). BMI and neuronal integrity in healthy, cognitively normal elderly: a proton magnetic resonance spectroscopy study. *Obesity* 18 743–748. 10.1038/oby.2009.325 19816410 PMC2847061

[B52] Gordon B. A. Rykhlevskaia E. I Brumback C. R. Lee Y. Elavsky S. Konopack J. F. (2008). Neuroanatomical correlates of aging, cardiopulmonary fitness level, and education. *Psychophysiology* 45 825–838. 10.1111/j.1469-8986.2008.00676.x 18627534 PMC5287394

[B53] Guiney H. Lucas S. J. Cotter J. D. Machado L. (2015). Evidence cerebral blood-flow regulation mediates exercise-cognition links in healthy young adults. *Neuropsychology* 29 1–9. 10.1037/neu0000124 25068671

[B54] Han X. Jovicich J. Salat D. van der Kouwe A. Quinn B. Czanner S. (2006). Reliability of MRI-derived measurements of human cerebral cortical thickness: the effects of field strength, scanner upgrade and manufacturer. *Neuroimage* 32 180–194. 10.1016/j.neuroimage.2006.02.051 16651008

[B55] Harris A. D. Glaubitz B. Near J. John Evans C. Puts N. A. Schmidt-Wilcke T. (2014). Impact of frequency drift on gamma-aminobutyric acid-edited MR spectroscopy. *Magn. Reson. Med.* 72 941–948. 10.1002/mrm.25009 24407931 PMC4017007

[B56] Harris A. D. Puts N. A. J. Edden R. A. E. (2015). Tissue correction for GABA-edited MRS: considerations of voxel composition, tissue segmentation, and tissue relaxations. *J. Magn. Reson. Imaging* 42 1431–1440. 10.1002/jmri.24903 26172043 PMC4615266

[B57] Hermans L. Levin O. Maes C. van Ruitenbeek P. Heise K. F. Edden R. A. E. (2018). GABA levels and measures of intracortical and interhemispheric excitability in healthy young and older adults: an MRS-TMS study. *Neurobiol. Aging* 65 168–177. 10.1016/j.neurobiolaging.2018.01.023 29494863

[B58] Heyn P. Abreu B. C. Ottenbacher K. J. (2004). The effects of exercise training on elderly persons with cognitive impairment and dementia: a meta-analysis. *Arch. Phys. Med. Rehabil.* 85 1694–1704. 10.1016/j.apmr.2004.03.019 15468033

[B59] Ho A. J. Raji C. A. Becker J. T. Lopez O. L. Kuller L. H. Hua X. (2011). The effects of physical activity, education, and body mass index on the aging brain. *Hum. Brain Mapp.* 32 1371–1382. 10.1002/hbm.21113 20715081 PMC3184838

[B60] Hu J. Yang S. Xuan Y. Jiang Q. Yang Y. Haacke E. M. (2007). Simultaneous detection of resolved glutamate, glutamine, and gamma-aminobutyric acid at 4 T. *J. Magn. Reson.* 185 204–213. 10.1016/j.jmr.2006.12.010 17223596 PMC1995429

[B61] Im K. Lee J.-M. Lyttelton O. Kim S. H. Evans A. C. Kim S. I. (2008). Brain size and cortical structure in the adult human brain. *Cereb. Cortex* 18 2181–2191. 10.1093/cercor/bhm244 18234686

[B62] Jenkinson M. Beckmann C. F. Behrens T. E. J. Woolrich M. W. Smith S. M. (2012). FSL. *Neuroimage* 62 782–790. 10.1016/j.neuroimage.2011.09.015 21979382

[B63] Johnson N. F. Kim C. Clasey J. L. Bailey A. Gold B. T. (2012). Cardiorespiratory fitness is positively correlated with cerebral white matter integrity in healthy seniors. *Neuroimage* 59 1514–1523. 10.1016/j.neuroimage.2011.08.032 21875674 PMC3230672

[B64] Jonasson L. S. Nyberg L. Kramer A. F. Lundquist A. Riklund K. Boraxbekk C. J. (2016). Aerobic exercise intervention, cognitive performance, and brain structure: results from the physical influences on brain in aging (PHIBRA) study. *Front. Aging Neurosci.* 8:336. 10.3389/fnagi.2016.00336 28149277 PMC5241294

[B65] Jovicich J. Czanner S. Greve D. Haley E. van der Kouwe A. Gollub R. (2006). Reliability in multi-site structural MRI studies: effects of gradient non-linearity correction on phantom and human data. *Neuroimage* 30 436–443. 10.1016/j.neuroimage.2005.09.046 16300968

[B66] Kalisch T. Ragert P. Schwenkreis P. Dinse H. R. Tegenthoff M. (2009). Impaired tactile acuity in old age is accompanied by enlarged hand representations in somatosensory cortex. *Cereb. Cortex* 19 1530–1538. 10.1093/cercor/bhn190 19008462

[B67] Kantak S. S. Stinear J. W. Buch E. R. Cohen L. G. (2012). Rewiring the brain: potential role of the premotor cortex in motor control, learning, and recovery of function following brain injury. *Neurorehabil. Neural Repair.* 26 282–292. 10.1177/1545968311420845 21926382 PMC4886541

[B68] Kantarci K. Senjem M. L. Avula R. Zhang B. Samikoglu A. R. Weigand S. D. (2011). Diffusion tensor imaging and cognitive function in older adults with no dementia. *Neurology* 77 26–34. 10.1212/WNL.0b013e31822313dc 21593440 PMC3127333

[B69] Kaup A. R. Mirzakhanian H. Jeste D. V. Eyler L. T. (2011). A Review of the brain structure correlates of successful Cognitive aging. *J. Neuropsychiatr. Clin. Neurosci.* 23 6–15. 10.1176/appi.neuropsych.23.1.6 PMC3068909 21304134

[B70] Kawagoe T. Onoda K. Yamaguchi S. (2017). Associations among executive function, cardiorespiratory fitness, and brain network properties in older adults. *Sci. Rep.* 7:40107. 10.1038/srep40107 28054664 PMC5215211

[B71] Kreis R. (2016). The trouble with quality filtering based on relative Cramér-Rao lower bounds. *Magn. Reson. Med.* 75 15–18. 10.1002/mrm.25568 25753153

[B72] Kukke S. N. Paine R. W. Chao C. C. de Campos A. C. Hallett M. (2014). Efficient and reliable characterization of the corticospinal system using transcranial magnetic stimulation. *J. Clin. Neurophysiol.* 31 246–252. 10.1097/WNP.0000000000000057 24887609 PMC4744647

[B73] Lulic T. El-Sayes J. Fassett H. J. Nelson A. J. (2017). Physical activity levels determine exercise-induced changes in brain excitability. *PLoS One* 12:e0173672. 10.1371/journal.pone.0173672 28278300 PMC5344515

[B74] Macsween A. (2001). The reliability and validity of the Astrand nomogram and linear extrapolation for deriving VO2max from submaximal exercise data. *J. Sports Med. Phys. Fitness* 41 312–317. 11533560

[B75] Maddock R. J. Casazza G. A. Fernandez D. H. Maddock M. I. (2016). Acute modulation of cortical glutamate and GABA content by physical activity. *J. Neurosci.* 36 2449–2457. 10.1523/JNEUROSCI.3455-15.2016 26911692 PMC6705493

[B76] Marks B. L. Katz L. M. Styner M. Smith J. K. (2011). Aerobic fitness and obesity: relationship to cerebral white matter integrity in the brain of active and sedentary older adults. *Br. J. Sports Med.* 45 1208–1215. 10.1136/bjsm.2009.068114 20558529

[B77] McCarthy C. S. Ramprashad A. Thompson C. Botti J.-A. Coman I. L. Kates W. R. (2015). A comparison of FreeSurfer-generated data with and without manual intervention. *Front. Neurosci.* 9:379. 10.3389/fnins.2015.00379 26539075 PMC4612506

[B78] McGregor K. M. Crosson B. Mammino K. Omar J. García P. S. Nocera J. R. (2017). Influences of 12-week physical activity interventions on TMS measures of cortical network inhibition and upper extremity motor performance in older adults-a feasibility study. *Front. Aging Neurosci.* 9:422. 10.3389/fnagi.2017.00422 29354049 PMC5758495

[B79] McGregor K. M. Heilman K. M. Nocera J. R. Patten C. Manini T. M. Crosson B. (2012). Aging, aerobic activity and interhemispheric communication. *Brain Sci.* 2 634–648. 10.3390/brainsci2040634 24961264 PMC4061818

[B80] McGregor K. M. Nocera J. R. Sudhyadhom A. Patten C. Manini T. M. Kleim J. A. (2013). Effects of aerobic fitness on aging-related changes of interhemispheric inhibition and motor performance. *Front. Aging Neurosci.* 5:66. 10.3389/fnagi.2013.00066 24198784 PMC3812779

[B81] McGregor K. M. Zlatar Z. Kleim E. Sudhyadhom A. Bauer A. Phan S. (2011). Physical activity and neural correlates of aging: a combined TMS/fMRI study. *Behav. Brain Res.* 222 158–168. 10.1016/j.bbr.2011.03.042 21440574 PMC3713467

[B82] Mescher M. Merkle H. Kirsch J. Garwood M. Gruetter R. (1998). Simultaneous in vivo spectral editing and water suppression. *NMR Biomed.* 11 266–272. 10.1002/(sici)1099-1492(199810)11:6<266::aid-nbm530>3.0.co;2-j 9802468

[B83] Mielke M. M. Vemuri P. Rocca W. A. (2014). Clinical epidemiology of Alzheimer’s disease: assessing sex and gender differences. *Clin. Epidemiol.* 6 37–48. 10.2147/clep.s37929 24470773 PMC3891487

[B84] Mikkelsen M. Barker P. B. Bhattacharyya P. K. Brix M. K. Buur P. F. Cecil K. M. (2017). Big GABA: edited MR spectroscopy at 24 research sites. *Neuroimage* 159 32–45. 10.1016/j.neuroimage.2017.07.021 28716717 PMC5700835

[B85] Molin C. J. Punga A. R. (2016). Compound motor action potential: electrophysiological marker for muscle training. *J. Clin. Neurophysiol.* 33 340–345. 10.1097/WNP.0000000000000252 26744834

[B86] Mooney R. A. Cirillo J. Byblow W. D. (2017). GABA and primary motor cortex inhibition in young and older adults: a multimodal reliability study. *J. Neurophysiol.* 118 425–433. 10.1152/jn.00199.2017 28424294 PMC5506262

[B87] Motulsky H. J. Brown R. E. (2006). Detecting outliers when fitting data with non-linear regression - a new method based on robust non-linear regression and the false discovery rate. *BMC Bioinformatics* 7:123. 10.1186/1471-2105-7-123 16526949 PMC1472692

[B88] Mullins P. G. McGonigle D. J. O’Gorman R. L. Puts N. A. Vidyasagar R. Evans C. J. (2014). Current practice in the use of MEGA-PRESS spectroscopy for the detection of GABA. *Neuroimage* 86 43–52. 10.1016/j.neuroimage.2012.12.004 23246994 PMC3825742

[B89] Nasreddine Z. S. Phillips N. A. Bedirian V. Charbonneau S. Whitehead V. Collin I. (2005). The montreal cognitive assessment, MoCA: a brief screening tool for mild cognitive impairment. *J. Am. Geriatr. Soc.* 53 695–699. 10.1111/j.1532-5415.2005.53221.x 15817019

[B90] Oberlin L. E. Verstynen T. D. Burzynska A. Z. Voss M. W. Prakash R. S. Chaddock-Heyman L. (2016). White matter microstructure mediates the relationship between cardiorespiratory fitness and spatial working memory in older adults. *Neuroimage* 131 91–101. 10.1016/j.neuroimage.2015.09.053 26439513 PMC4826637

[B91] Oldfield R. C. (1971). The assessment and analysis of handedness: the Edinburgh inventory. *Neuropsychologia* 9 97–113. 10.1016/0028-3932(71)90067-4 5146491

[B92] Persky R. W. Turtzo L. C. McCullough L. D. (2010). Stroke in women: disparities and outcomes. *Curr. Cardiol. Rep.* 12 6–13. 10.1007/s11886-009-0080-2 20425178 PMC2861793

[B93] Pescatello L. S. Ross A. Deborah R. Thompson P. D. (2014). *ACSM’s Guidelines for Exercise Testing and Prescription 9th Ed. 2014*, 9th Edn Philadelphia, PA: CCA.

[B94] Provencher S. W. (2001). Automatic quantitation of localized in vivo 1H spectra with LCModel. *NMR Biomed.* 14 260–264. 10.1002/nbm.698 11410943

[B95] R Core Team (2018). *R: A Language and Environment for Statistical Computing*. Vienna: R Foundation for Statistical Computing.

[B96] Reuter M. Rosas H. D. Fischl B. (2010). Highly accurate inverse consistent registration: a robust approach. *Neuroimage* 53 1181–1196. 10.1016/j.neuroimage.2010.07.020 20637289 PMC2946852

[B97] Reuter M. Schmansky N. J. Rosas H. D. Fischl B. (2012). Within-subject template estimation for unbiased longitudinal image analysis. *Neuroimage* 61 1402–1418. 10.1016/j.neuroimage.2012.02.084 22430496 PMC3389460

[B98] Rossini P. M. Burke D. Chen R. Cohen L. G. Daskalakis Z. Di Iorio R. (2015). Non-invasive electrical and magnetic stimulation of the brain, spinal cord, roots and peripheral nerves: basic principles and procedures for routine clinical and research application. An updated report from an I.F.C.N. Committee. *Clin. Neurophysiol.* 126 1071–1107. 10.1016/j.clinph.2015.02.001 25797650 PMC6350257

[B99] Rueckert D. Sonoda L. I. Hayes C. Hill D. L. G. Leach M. O. Hawkes D. J. (1999). Non-rigid registration using free-form deformations: application to breast MR images. *IEEE Trans. Med. Imaging* 18 712–721. 10.1109/42.796284 10534053

[B100] Sanaei Nezhad F. Anton A. Michou E. Jung J. Y. Parkes L. M. Williams S. R. (2018). Quantification of GABA, glutamate and glutamine in a single measurement at 3 T using GABA-edited MEGA-PRESS. *NMR Biomed.* 31:e3847. 10.1002/nbm.3847 29130590 PMC5765428

[B101] Schambra H. M. Ogden R. T. Martinez-Hernandez I. E. Lin X. Chang Y. B. Rahman A. (2015). The reliability of repeated TMS measures in older adults and in patients with subacute and chronic stroke. *Front. Cell Neurosci.* 9:335. 10.3389/fncel.2015.00335 26388729 PMC4555014

[B102] Schulz R. Zimerman M. Timmermann J. E. Wessel M. J. Gerloff C. Hummel F. C. (2014). White matter integrity of motor connections related to training gains in healthy aging. *Neurobiol. Aging* 35 1404–1411. 10.1016/j.neurobiolaging.2013.11.024 24387983

[B103] Ségonne F. Dale A. M. Busa E. Glessner M. Salat D. Hahn H. K. (2004). A hybrid approach to the skull stripping problem in MRI. *Neuroimage* 22 1060–1075. 10.1016/j.neuroimage.2004.03.032 15219578

[B104] Seidler R. D. Bernard J. A. Burutolu T. B. Fling B. W. Gordon M. T. Gwin J. T. (2010). Motor control and aging: links to age-related brain structural, functional, and biochemical effects. *Neurosci. Biobehav. Rev.* 34 721–733. 10.1016/j.neubiorev.2009.10.005 19850077 PMC2838968

[B105] Sheffield P. Noseworthy M. Bienenstock J. (2009). Simultaneous quantification of (-aminobutyric acid, glutamate, and glutamine at 3T. *Int. Soc. Magn. Reson. Medicine.* 17:2399.

[B106] Siconolfi S. F. Cullinane E. M. Carleton R. A. Thompson P. D. (1982). Assessing VO2max in epidemiologic studies: modification of the Astrand-Rhyming test. *Med. Sci. Sports Exerc.* 14 335–338. 7154887

[B107] Smith S. M. (2002). Fast robust automated brain extraction. *Hum. Brain Mapp.* 17 143–155. 10.1002/hbm.10062 12391568 PMC6871816

[B108] Smith S. M. Jenkinson M. Johansen-Berg H. Rueckert D. Nichols T. E. Mackay C. E. (2006). Tract-based spatial statistics: Voxelwise analysis of multi-subject diffusion data. *Neuroimage* 31 1487–1505. 10.1016/j.neuroimage.2006.02.024 16624579

[B109] Soares J. M. Marques P. Alves V. Sousa N. (2013). A hitchhiker’s guide to diffusion tensor imaging. *Front. Neurosci.* 7:31. 10.3389/fnins.2013.00031 23486659 PMC3594764

[B110] Sridharan A. Willette A. A. Bendlin B. B. Alexander A. L. Coe C. L. Voytko M. L. (2012). Brain volumetric and microstructural correlates of executive and motor performance in aged rhesus monkeys. *Front. Aging Neurosci.* 4:31. 10.3389/fnagi.2012.00031 23162464 PMC3492760

[B111] Stagg C. J. Bestmann S. Constantinescu A. O. Moreno L. M. Allman C. Mekle R. (2011). Relationship between physiological measures of excitability and levels of glutamate and GABA in the human motor cortex. *J. Physiol.* 589 5845–5855. 10.1113/jphysiol.2011.216978 22005678 PMC3249054

[B112] Sullivan E. V. Rohlfing T. Pfefferbaum A. (2010). Quantitative fiber tracking of lateral and interhemispheric white matter systems in normal aging: relations to timed performance. *Neurobiol. Aging* 31 464–481. 10.1016/j.neurobiolaging.2008.04.007 18495300 PMC2815144

[B113] Tremblay S. Beaule V. Proulx S. de Beaumont L. Marjanska M. Doyon J. (2013). Relationship between transcranial magnetic stimulation measures of intracortical inhibition and spectroscopy measures of GABA and glutamate glutamine. *J. Neurophysiol.* 109 1343–1349. 10.1152/jn.00704.2012 23221412 PMC3602833

[B114] Tseng B. Y. Gundapuneedi T. Khan M. A. Diaz-Arrastia R. Levine B. D. Lu H. (2013). White matter integrity in physically fit older adults. *Neuroimage* 82 510–516. 10.1016/j.neuroimage.2013.06.011 23769914 PMC3759589

[B115] Turco C. V. Locke M. B. El-Sayes J. Tommerdahl M. Nelson A. J. (2018). Exploring behavioral correlates of afferent inhibition. *Brain Sci.* 8:E64. 10.3390/brainsci8040064 29641439 PMC5924400

[B116] Wang X. Casadio M. Weber K. A. Mussa-Ivaldi F. A. Parrish T. B. Parrish T. B. (2014). White matter microstructure changes induced by motor skill learning utilizing a body machine interface. *Neuroimage* 88 32–40. 10.1016/j.neuroimage.2013.10.066 24220038 PMC4016193

[B117] Warburton D. E. R. Bredin S. S. D. (2016). Reflections on physical activity and health: what should we recommend? *Can. J. Cardiol.* 32 495–504. 10.1016/j.cjca.2016.01.024 26995692

[B118] Weinstein A. M. Voss M. W. Prakash R. S. Chaddock L. Szabo A. White S. M. (2012). The association between aerobic fitness and executive function is mediated by prefrontal cortex volume. *Brain Behav. Immun.* 26 811–819. 10.1016/j.bbi.2011.11.008 22172477 PMC3321393

[B119] Weuve J. Kang J. H. Manson J. E. Breteler M. M. Ware J. H. Grodstein F. (2004). Physical activity, including walking, and cognitive function in older women. *JAMA* 292 1454–1461. 10.1001/jama.292.12.1454 15383516

[B120] Williams V. J. Hayes J. P. Forman D. E. Salat D. H. Sperling R. A. Verfaellie M. (2017). Cardiorespiratory fitness is differentially associated with cortical thickness in young and older adults. *Neuroimage* 146 1084–1092. 10.1016/j.neuroimage.2016.10.033 27989841 PMC5321861

[B121] Winkler A. M. Kochunov P. Blangero J. Almasy L. Zilles K. Fox P. T. (2010). Cortical thickness or grey matter volume? The importance of selecting the phenotype for imaging genetics studies. *Neuroimage* 53 1135–1146. 10.1016/j.neuroimage.2009.12.028 20006715 PMC2891595

[B122] Wood K. N. Nikolov R. Shoemaker J. K. (2016). Impact of long-term endurance training vs. guideline-based physical activity on brain structure in healthy aging. *Front. Aging Neurosci.* 8:155. 10.3389/fnagi.2016.00155 27445798 PMC4928447

[B123] Yaffe K. Barnes D. Nevitt M. Lui L. Y. Covinsky K. (2001). A prospective study of physical activity and cognitive decline in elderly women: women who walk. *Arch. Int. Med.* 161 1703–1708. 11485502 10.1001/archinte.161.14.1703

[B124] Yousry T. A. Schmid U. D. Alkadhi H. Schmidt D. Peraud A. Buettner A. (1997). Localization of the motor hand area to a knob on the precentral gyrus. A new landmark. *Brain* 120 141–157. 10.1093/brain/120.1.141 9055804

