# Supplementary material for: Fitness Level Influences White Matter Microstructure in Postmenopausal Women
Source: Front Aging Neurosci. 2020 May 29;12:129. doi: 10.3389/fnagi.2020.00129 (PMC7273967; doi:10.3389/fnagi.2020.00129)
Supplement: Supplementary file 1 [file Table_1.docx]

| ***Supplementary Table 1: Results of multiple linear regression of FA in the sensorimotor area and reference ROIs against fitness and age.*** | | | | | | |
| --- | --- | --- | --- | --- | --- | --- |
|  |  | ***Coefficients (β)*** | ***SE*** | ***95% CI*** | *p-value* | *R^2^  (Adj.)* |
| ***M1*** | *VO_2max_^ADJ^* | 0.04 | 0.02 | -0.01 : 0.1 | 0.09 | 0.16 |
|  | *Age* | -0.2 | 0.1 | -0.4 : 0.03 | 0.09 |  |
| ***PMv*** | *VO_2max_^ADJ^* | 0.02 | 0.02 | -0.03 : 0.1 | 0.33 | 0.06 |
|  | *Age* | -0.2 | 0.1 | -0.4 : 0.1 | 0.14 |  |
| ***PMd*** | *VO_2max_^ADJ^* | 0.1 | 0.02 | 0.02 : 0.1 | **0.01** | 0.26 |
|  | *Age* | -0.1 | 0.1 | -0.4 : 0.1 | 0.24 |  |
| ***SMA*** | *VO_2max_^ADJ^* | 0.03 | 0.03 | -0.03 :0.1 | 0.30 | 0.08 |
|  | *Age* | -0.1 | 0.1 | -0.4 : 0.2 | 0.42 |  |
| ***preSMA*** | *VO_2max_^ADJ^* | 0.0 | 0.0 | -0.02 : 0.1 | 0.767 | 0.12 |
|  | *Age* | -0.2 | 0.1 | -0.4 :0.01 | 0.151 |  |
| ***S1*** | *VO_2max_^ADJ^* | 0.03 | 0.02 | -0.01 : 0.1 | 0.15 | 0.13 |
|  | *Age* | -0.2 | 0.1 | -0.4 : 0.03 | 0.10 |  |
| ***CC Genu*** | *VO_2max_^ADJ^* | 0.05 | 0.04 | -0.03 : 0.1 | 0.20 | 0.05 |
|  | *Age* | -0.2 | 0.2 | -0.6 : 0.2 | 0.23 |  |
| ***CC Body*** | *VO_2max_^ADJ^* | 0.01 | 0.05 | -0.1 : 0.1 | 0.78 | 0.01 |
|  | *Age* | -0.1 | 0.2 | -0.5 : 0.4 | 0.70 |  |
| ***CC Splenium*** | *VO_2max_^ADJ^* | 0.03 | 0.02 | -0.02 : 0.1 | 0.29 | 0.12 |
|  | *Age* | -0.2 | 0.1 | -0.4 : 0.1 | 0.06 |  |
| ***Anterior CR*** | *VO_2max_^ADJ^* | -0.03 | 0.03 | -0.1 : 0.04 | 0.41 | 0.04 |
|  | *Age* | -0.06 | 0.1 | -0.3 : 0.2 | 0.65 |  |
| ***Superior CR*** | *VO_2max_^ADJ^* | <0.01 | 0.02 | -0.04 : 0.1 | 0.86 | 0.05 |
|  | *Age* | -0.1 | 0.1 | -0.3 : 0.1 | 0.30 |  |
| ***Posterior CR*** | *VO_2max_^ADJ^* | 0.01 | 0.03 | -0.05 : 0.1 | 0.75 | 0.004 |
|  | *Age* | <0.01 | 0.1 | -0.3 : 0.3 | 0.97 |  |
| ***Cingulum Hippocampus*** | *VO_2max_^ADJ^* | 0.05 | 0.03 | -0.01 : 0.1 | 0.10 | 0.07 |
|  | *Age* | -0.1 | 0.1 | -0.5 : 0.2 | 0.35 |  |
| ***Cingulum Cingulate Gyrus*** | *VO_2max_^ADJ^* | 0.03 | 0.03 | -0.04 : 0.1 | 0.38 | 0.11 |
|  | *Age* | -0.3 | 0.2 | -0.7 : 0.01 | 0.06 |  |
| ***SLF*** | *VO_2max_^ADJ^* | <0.01 | 0.02 | -0.02 : 0.1 | 0.85 | 0.05 |
|  | *Age* | -0.2 | 0.1 | -0.4 : 0.1 | 0.09 |  |
| *Bolded values indicate significance as shown. Coefficient, error and confidence interval values are x10^-2^.* | | | | | | |
