# Supplementary material for: Fitness Level Influences White Matter Microstructure in Postmenopausal Women
Source: Front Aging Neurosci. 2020 May 29;12:129. doi: 10.3389/fnagi.2020.00129 (PMC7273967; doi:10.3389/fnagi.2020.00129)
Supplement: Supplementary file 2 [file Table_2.docx]

| ***Supplementary Table 2: Results of multiple linear regression of RD in the sensorimotor area and reference ROIs against fitness and age.*** | | | | | | |
| --- | --- | --- | --- | --- | --- | --- |
|  |  | ***Coefficients (β)*** | ***SE*** | ***95% CI*** | *p-value* | *R^2^  (Adj.)* |
| ***M1*** | *VO_2max_^ADJ^* | -2.53 | 2.48 | -7.68 : 2.63 | 0.32 | 0.13 |
|  | *Age* | 23.78 | 11.24 | 0.41 : 47.15 | 0.05 |  |
| ***PMv*** | *VO_2max_^ADJ^* | -1.5 | 3.13 | -8.10 : 4.94 | 0.62 | 0.04 |
|  | *Age* | 23.48 | 14.21 | -6.07 : 53.04 | 0.11 |  |
| ***PMd*** | *VO_2max_^ADJ^* | -4.46 | 2.64 | -9.97 : 1.03 | 0.11 | 0.17 |
|  | *Age* | 21.75 | 11.15 | -1.52 : 45.02 | 0.07 |  |
| ***SMA*** | *VO_2max_^ADJ^* | -2.50 | 3.73 | -10.26 : 5.25 | 0.51 | 0.05 |
|  | *Age* | 27.99 | 16.92 | -7.19 : 63.16 | 0.11 |  |
| ***preSMA*** | *VO_2max_^ADJ^* | -3.51 | 3.28 | -10.32 : 3.30 | 0.30 | 0.16 |
|  | *Age* | 33.70 | 14.85 | 2.82 : 64.68 | **0.03** |  |
| ***S1*** | *VO_2max_^ADJ^* | -0.40 | 2.38 | -5.34 : 4.54 | 0.87 | 0.13 |
|  | *Age* | 24.98 | 10.78 | 2.57 : 47.39 | **0.03** |  |
| ***CC Genu*** | *VO_2max_^ADJ^* | -7.12 | 5.72 | -19.01 : 4.78 | 0.23 | 0.08 |
|  | *Age* | 41.25 | 25.94 | -12.70 : 95.19 | 0.13 |  |
| ***CC Body*** | *VO_2max_^ADJ^* | 0.59 | 7.39 | -14.78 : 15.96 | 0.94 | 0.04 |
|  | *Age* | 31.44 | 33.53 | -38.30 : 101.16 | 0.36 |  |
| ***CC Splenium*** | *VO_2max_^ADJ^* | 0.77 | 3.52 | -8.08 : 6.54 | 0.83 | 0.05 |
|  | *Age* | 28.24 | 15.95 | -4.93 : 61.41 | 0.09 |  |
| ***Anterior CR*** | *VO_2max_^ADJ^* | 2.81 | 4.19 | -5.89 : 11.52 | 0.51 | 0.03 |
|  | *Age* | 8.68 | 18.99 | -30 .81 : 48.16 | 0.65 |  |
| ***Superior CR*** | *VO_2max_^ADJ^* | -0.58 | 3.18 | -7.20 : 6.04 | 0.86 | 0.04 |
|  | *Age* | 13.68 | 14.44 | -16.35 : 43.71 | 0.35 |  |
| ***Posterior CR*** | *VO_2max_^ADJ^* | 1.99 | 4.12 | -6.57 : 10.54 | 0.63 | 0.01 |
|  | *Age* | 0.33 | 18.66 | -38.48 : 39.13 | 0.99 |  |
| ***Cingulum Hippocampus*** | *VO_2max_^ADJ^* | 2.39 | 3.58 | -5.05 : 9.83 | 0.51 | 0.02 |
|  | *Age* | 23.82 | 16.22 | -9.92 : 57.56 | 0.16 |  |
| ***Cingulum Cingulate Gyrus*** | *VO_2max_^ADJ^* | 0.66 | 4.74 | -9.19 : 10.51 | 0.89 | 0.15 |
|  | *Age* | 53.41 | 21.48 | 8.73 : 98.08 | **0.02** |  |
| ***SLF*** | *VO_2max_^ADJ^* | 0.02 | 3.24 | -6.72 : 6.75 | 0.99 | 0.05 |
|  | *Age* | 26.57 | 14.69 | -39.83 : 57.12 | 0.08 |  |
| *One outlier removed from PMd. Bolded values indicate significance as shown. Coefficient, error and confidence interval values are x10^-7^.* | | | | | | |
