# Supplementary material for: Fitness Level Influences White Matter Microstructure in Postmenopausal Women
Source: Front Aging Neurosci. 2020 May 29;12:129. doi: 10.3389/fnagi.2020.00129 (PMC7273967; doi:10.3389/fnagi.2020.00129)
Supplement: Supplementary file 3 [file Table_3.docx]

| ***Supplementary Table 3: Results of multiple linear regression of AD in the sensorimotor area and reference ROIs against fitness and age.*** | | | | | | |
| --- | --- | --- | --- | --- | --- | --- |
|  |  | ***Coefficients (β)*** | ***SE*** | ***95% CI*** | *p-value* | *R^2^  (Adj.)* |
| ***M1*** | *VO_2max_^ADJ^* | 6.16 | 3.45 | -1.01 : 13.34 | 0.09 | 0.06 |
|  | *Age* | -8.71 | 15.66 | -41.13 : 23.85 | 0.58 |  |
| ***PMv*** | *VO_2max_^ADJ^* | 3.01 | 3.73 | -4.75 : 10.77 | 0.43 | 0.03 |
|  | *Age* | -3.39 | 16.93 | -38.58 : 31.81 | 0.84 |  |
| ***PMd*** | *VO_2max_^ADJ^* | 2.86 | 5.57 | -8.73 : 14.45 | 0.61 | 0.03 |
|  | *Age* | 15.25 | 25.27 | -37.30 : 67.81 | 0.55 |  |
| ***SMA*** | *VO_2max_^ADJ^* | 2.19 | 4.09 | -6.33 : 10.69 | 0.60 | 0.07 |
|  | *Age* | 21.47 | 18.56 | -17.13 : 60.07 | 0.26 |  |
| ***preSMA*** | *VO_2max_^ADJ^* | 1.57 | 5.26 | -9.36 : 12.50 | 0.77 | 0.02 |
|  | *Age* | 14.87 | 23.83 | -34.68 : 64.43 | 0.54 |  |
| ***S1*** | *VO_2max_^ADJ^* | 8.24 | 3.76 | 0.42 : 16.06 | **0.04** | 0.11 |
|  | *Age* | -27.75 | 17.06 | -38.25 : 32.70 | 0.87 |  |
| ***CC Genu*** | *VO_2max_^ADJ^* | 0.72 | 5.43 | -10.58 : 12.02 | 0.90 | 0.06 |
|  | *Age* | 27.16 | 24.64 | -24.08 : 78.41 | 0.28 |  |
| ***CC Body*** | *VO_2max_^ADJ^* | 6.61 | 6.27 | -6.43 : 19.64 | 0.30 | 0.07 |
|  | *Age* | 46.40 | 28.42 | -12.70 : 10.55 | 0.11 |  |
| ***CC Splenium*** | *VO_2max_^ADJ^* | 11.36 | 6.05 | -1.22 : 23.93 | 0.07 | 0.06 |
|  | *Age* | 7.35 | 27.43 | -49.68 : 64.39 | 0.79 |  |
| ***Anterior CR*** | *VO_2max_^ADJ^* | -1.03 | 3.85 | -9.03 : 6.97 | 0.79 | 0.01 |
|  | *Age* | 4.60 | 17.46 | -31.70 : 40.90 | 0.80 |  |
| ***Superior CR*** | *VO_2max_^ADJ^* | -1.34 | 4.34 | -10.36 : 7.68 | 0.76 | 0.02 |
|  | *Age* | -12.78 | 19.67 | -53.69 : 28.14 | 0.52 |  |
| ***Posterior CR*** | *VO_2max_^ADJ^* | 5.69 | 6.12 | -7.03 : 18.41 | 0.36 | 0.06 |
|  | *Age* | -9.65 | 27.74 | -67.34 : 48.05 | 0.73 |  |
| ***Cingulum Hippocampus*** | *VO_2max_^ADJ^* | 19.19 | 6.63 | 5.40 : 32.98 | **0.01** | 0.22 |
|  | *Age* | -3.84 | 30.07 | -66.38 : 58.70 | 0.90 |  |
| ***Cingulum Cingulate Gyrus*** | *VO_2max_^ADJ^* | 7.33 | 7.32 | -7.90 : 22.55 | 0.22 | 0.06 |
|  | *Age* | 17.51 | 33.20 | -51.52 : 86.54 | 0.60 |  |
| ***SLF*** | *VO_2max_^ADJ^* | -3.75 | 4.47 | -13.05 : 5.54 | 0.41 | 0.03 |
|  | *Age* | -4.96 | 20.27 | -47.12 : 37.20 | 0.81 |  |
| *Bolded values indicate significance as shown. Coefficient, error and confidence interval values are x10^-7^.* | | | | | | |
