# Supplementary material for: Fitness Level Influences White Matter Microstructure in Postmenopausal Women
Source: Front Aging Neurosci. 2020 May 29;12:129. doi: 10.3389/fnagi.2020.00129 (PMC7273967; doi:10.3389/fnagi.2020.00129)
Supplement: Supplementary file 4 [file Table_4.docx]

| ***Supplementary Table 4: Results of multiple linear regression of MD in the sensorimotor area and reference ROIs against fitness and age.*** | | | | | | |
| --- | --- | --- | --- | --- | --- | --- |
|  |  | ***Coefficients (β)*** | ***SE*** | ***95% CI*** | *p-value* | *R^2^  (Adj.)* |
| ***M1*** | *VO_2max_^ADJ^* | 0.28 | 1.91 | -3.69 : 4.26 | 0.88 | 0.01 |
|  | *Age* | 13.19 | 8.67 | -4.85 : 31.22 | 0.14 |  |
| ***PMv*** | *VO_2max_^ADJ^* | -0.18 | 2.88 | -6.17 : 5.81 | 0.95 | 0.05 |
|  | *Age* | 14.32 | 13.06 | -12.84 : 41.48 | 0.29 |  |
| ***PMd*** | *VO_2max_^ADJ^* | -0.32 | 2.63 | -5.81 : 5.16 | 0.90 | 0.01 |
|  | *Age* | 16.78 | 11.10 | -6.38 : 39.94 | 0.15 |  |
| ***SMA*** | *VO_2max_^ADJ^* | -0.95 | 3.18 | -7.57 : 5.67 | 0.77 | 0.06 |
|  | *Age* | 26.27 | 14.43 | -3.74 : 56.29 | 0.08 |  |
| ***preSMA*** | *VO_2max_^ADJ^* | -1.98 | 3.52 | -9.31 : 5.34 | 0.58 | 0.06 |
|  | *Age* | 28.47 | 15.97 | -47.28 : 61.67 | 0.09 |  |
| ***S1*** | *VO_2max_^ADJ^* | 2.26 | 2.16 | -2.23 : 6.75 | 0.31 | 0.07 |
|  | *Age* | 16.03 | 9.80 | -4.34 : 36.39 | 0.12 |  |
| ***CC Genu*** | *VO_2max_^ADJ^* | -4.48 | 4.73 | -14.32 : 5.37 | 0.36 | 0.07 |
|  | *Age* | 35.93 | 21.47 | -8.71 : 80.57 | 0.11 |  |
| ***CC Body*** | *VO_2max_^ADJ^* | 3.62 | 4.19 | -5.13 : 12.35 | 0.40 | 0.07 |
|  | *Age* | 17.70 | 19.39 | -22.75 : 58.15 | 0.37 |  |
| ***CC Splenium*** | *VO_2max_^ADJ^* | 3.09 | 3.64 | -4.47 : 10.65 | 0.41 | 0.01 |
|  | *Age* | 21.07 | 16.49 | -13.23 : 55.36 | 0.22 |  |
| ***Anterior CR*** | *VO_2max_^ADJ^* | 1.57 | 3.66 | -6.04 : 9.19 | 0.67 | 0.02 |
|  | *Age* | 7.13 | 16.60 | -27.40 : 41.66 | 0.67 |  |
| ***Superior CR*** | *VO_2max_^ADJ^* | -0.73 | 3.17 | -7.31 : 5.85 | 0.82 | 0.01 |
|  | *Age* | 5.68 | 14.35 | -24.17 : 35.52 | 0.70 |  |
| ***Posterior CR*** | *VO_2max_^ADJ^* | 3.25 | 4.34 | -5.78 : 12.27 | 0.46 | 0.03 |
|  | *Age* | -3.43 | 19.69 | -44.37 : 37.51 | 0.86 |  |
| ***Cingulum Hippocampus*** | *VO_2max_^ADJ^* | 8.00 | 3.97 | -0.02 : 16.02 | 0.05 | 0.12 |
|  | *Age* | 15.22 | 17.48 | -21.13 : 51.57 | 0.39 |  |
| ***Cingulum Cingulate Gyrus*** | *VO_2max_^ADJ^* | 2.82 | 4.74 | -7.04 : 12.67 | 0.56 | 0.09 |
|  | *Age* | 42.27 | 21.50 | -2.43 : 86.98 | 0.06 |  |
| ***SLF*** | *VO_2max_^ADJ^* | -1.15 | 3.07 | -7.54 : 5.24 | 0.71 | 0.07 |
|  | *Age* | 16.06 | 13.94 | -12.92 : 45.04 | 0.26 |  |
| *One outlier removed from PMd and CC Body. Coefficient, error and confidence interval values are x10^-7^.* | | | | | | |
